# A major and stable QTL confers impatiens necrotic spot virus resistance in lettuce cv. Eruption

**DOI:** 10.1007/s00122-025-05058-9

**Published:** 2025-11-28

**Authors:** Santosh Nayak, Kelley L. Richardson, Renée L. Eriksen, Daniel K. Hasegawa, William M. Wintermantel, Manoj Sapkota, Xuemei Tang, Shufen Chen, Meng Lin, Dongyan Zhao, Craig T. Beil, Moira J. Sheehan, Ivan Simko

**Affiliations:** 1 U.S. Department of Agriculture, Agricultural Research Service, Sam Farr United States Crop Improvement and Protection Research Center, 1636 East Alisal Street, Salinas, CA 93905 USA; 2 Breeding Insight, Cornell University, 525 Tower Road, Ithaca, NY 14853 USA; 3 Present Address: Department of Horticulture, University of Kentucky, 1405 Veterans Dr, Lexington, KY 40546 USA

## Abstract

**Key message:**

A large effect and environmentally stable QTL was identified on LG2 that confers high levels of INSV resistance in lettuce cultivar Eruption.

**Abstract:**

Impatiens necrotic spot virus (INSV) has recently emerged as a major threat to lettuce production in the Salinas Valley of California, the region which contributes over 60% of the US national supply. This thrips-transmitted virus can infect lettuce plants at any growth stage, causing premature death or a total loss of marketability. Both INSV and its thrips vector have broad host ranges, which complicate disease management. Utilizing genetic resistance is the most sustainable approach; however, complete immunity has not been identified and the genetic basis of resistance to INSV in lettuce remains poorly understood. This study aimed to identify quantitative trait loci (QTL) and elucidate the underlying mechanism of INSV resistance in ‘Eruption,’ a lettuce cultivar exhibiting highly stable partial resistance across environments. Using 162 F_6:8_ recombinant inbred lines (RILs) developed from a cross between moderately susceptible ‘Reine des Glaces’ and ‘Eruption,’ and a genetic linkage map comprising 1598 single nucleotide polymorphism (SNP) markers, phenotypic data collected from field and greenhouse experiments consistently revealed a highly significant, major QTL on linkage group 2. This QTL exhibited partial dominance with additive effects, explaining up to 61% of the total phenotypic variation for INSV disease severity. Furthermore, INSV resistance was found to be highly heritable, with heritability estimates of up to 0.89, indicating strong genetic control. Results of this study are crucial for fine mapping and the development of marker-assisted selection assays to accelerate the breeding of more advanced INSV-resistant lettuce cultivars.

**Supplementary Information:**

The online version contains supplementary material available at 10.1007/s00122-025-05058-9.

## Introduction

Lettuce (*Lactuca sativa* L.) is one of the most valuable agricultural commodities in California, ranking fourth in 2023 with an estimated revenue of approximately $4 billion (California Department of Food and Agriculture 2023). The state produces over 75% of the nation’s lettuce, with the Salinas Valley alone contributing over 60% of the US output to supply domestic and international markets (California Department of Food and Agriculture 2023). Lettuce grown in the Salinas Valley belongs to several horticultural types, including crisphead (iceberg), romaine (cos), looseleaf (green and red), Latin, Batavia, butterhead, and is cultivated year-round in open fields (except for a 2-week lettuce-free period from December 7 to 21 to manage lettuce mosaic virus under the host-free and disease control programs) (Smith et al. 2024; https://www.countyofmonterey.gov/).

Impatiens necrotic spot virus (INSV; family *Tospoviridae*, genus *Orthotospovirus*) causes substantial damage to lettuce and is transmitted by western flower thrips (*Frankliniella occidentalis*) which are small insects about 1–2 mm in length. INSV was first detected on lettuce crops in the Salinas Valley in 2006 (Koike et al. 2008) and has more recently emerged in winter lettuce production areas of Southern California and Arizona (Hasegawa et al. 2022). While its presence was limited to isolated outbreaks in a few commercial fields until 2017 in the Salinas Valley (Kuo et al. 2014), the disease has intensified in recent years, causing extensive and devastating losses across entire growing regions (Hasegawa and Del Pozo-Valdivia 2023; Simko et al. 2023a; Richardson et al. 2024). In 2022, outbreaks of INSV caused an estimated $150 million in economic losses to the lettuce industry in the Salinas Valley (Hsu 2023).

Lettuce plants infected with INSV exhibit a range of symptoms that vary in severity depending on cultivar, timing of infection, and environmental conditions. Common symptoms include appearance of tan to dark brown necrosis on the leaf and along the midrib, stunted growth, and deformities of heads (Fig. S1) that render them unmarketable (Hasegawa and Del Pozo-Valdivia 2023; Richardson et al. 2024). In severe cases, infected plants succumb to premature death. Infection and symptom expression can occur at any stage of plant development, but early infection is particularly detrimental.

INSV infects a diverse array of vegetables, ornamentals, and weeds that are common in the Salinas Valley, and can serve as reservoirs from which the virus can be acquired by thrips and transmitted to lettuce crops (Kuo et al. 2014). The broad host range of both INSV and thrips complicates disease management strategies. A lack of highly resistant lettuce cultivars, combined with the limited efficacy of available control measures for thrips, continues to make area-wide management of INSV challenging (Hasegawa and Del Pozo-Valdivia 2023). Enhancing genetic resistance through germplasm improvement offers a sustainable path forward to minimizing production losses associated with the disease.

Only a few lettuce cultivars with effective resistance to INSV, such as ‘Farmin RZ’ and ‘Jammin RZ’ (Rijk Zwaan, The De Lier, Netherlands), are currently available; however, patent protections (Schut et al. 2016) limit their accessibility for use in public and private breeding programs. To overcome this limitation, the United States Department of Agriculture-Agricultural Research Service (USDA-ARS) is actively developing INSV-resistant germplasm that is publicly available (Simko et al. 2018, 2023a; Richardson et al. 2024). These efforts have identified promising resistant germplasm, including the red Latin-type cultivar Eruption. Across multiple years of testing more than 1,000 accessions, ‘Eruption’ consistently exhibited one of the highest levels of resistance (Simko et al. 2018, 2023a), highlighting its potential for developing new resistant cultivars (Richardson et al. 2024). Generally, red leaf cultivars like Eruption were less affected by INSV compared to green leaf types (Simko et al. 2023a; Richardson et al. 2024), leading to the hypothesis that anthocyanins might be linked to this resistance. Anthocyanins, water-soluble flavonoids responsible for red and purple pigmentation in plants, including lettuce (Simko 2020; Su et al. 2020; Shi et al. 2022), are known to provide protection against various biotic and abiotic stresses (Steyn et al. 2002; Gould 2004). Nevertheless, the specific role of anthocyanins in conferring INSV resistance in lettuce remains poorly understood. While germplasm evaluations and genome-wide association studies (GWAS) have identified INSV-resistant lettuce germplasm and associated loci (Simko et al. 2018, 2023a; Richardson et al. 2024), the detailed genetic basis of this resistance remains largely unknown, limiting breeding progress. Identifying environmentally stable quantitative trait loci (QTL) and developing molecular assays for marker-assisted selection (MAS) are crucial for expediting the breeding process by improving selection efficiency. QTL mapping provides an opportunity to dissect the genetic architecture and identify functional alleles present in superior cultivars (Würschum 2012).

This study, building upon our previous findings (Simko et al. 2018, 2023a; Richardson et al. 2024), aims to identify stable QTL conferring INSV resistance using a recombinant inbred line (RIL) population developed from a cross between the moderately susceptible ‘Reine des Glaces’ and the partially resistant ‘Eruption’ (Fig. S1). We further investigate the relationships between anthocyanin content, INSV severity, and other morphological traits to assess potential genetic linkage and pleiotropic effects between these traits. This research will provide new insights by advancing our understanding of the genetic factors governing INSV resistance in lettuce.

## Materials and methods

### Plant materials

A mapping population comprising of 163 F_6:8_ recombinant inbred lines (RILs) was derived from a cross between ‘Reine des Glaces’ (PI 634668) and ‘Eruption’ (PI 613577). ‘Reine des Glaces,’ a Batavia-type lettuce with glabrous green lamina and undulated leaf margins, is moderately susceptible to INSV. ‘Eruption,’ a Latin-type lettuce with glabrous red lamina and entire leaf margins, possess a high degree of environmentally stable INSV resistance (Simko et al. 2018, 2023a; Richardson et al. 2024). This ‘Reine des Glaces × Eruption’ RIL mapping population (hereafter interchangeably referred to as B×E RILs) was originally developed for genetic analysis of resistance to lettuce drop (caused by *Sclerotinia minor*), and the methodology of its development is described in Mamo et al. (2019). Out of 163 RILs, one line, ‘B×E16-009,’ was not used in phenotyping due to seed shortage. An INSV-susceptible check, romaine-type cultivar ‘Defender’ (PI 667691), was included in each experiment.

### Phenotyping for INSV resistance

#### Field experiments

Three field experiments were conducted at the USDA Spence Research Farm located near Salinas in California (GPS location: 36.629046, − 121.539598) to evaluate the B×E RILs for INSV resistance under natural infection conditions. The first experiment was planted on 24 August 2022. The other two experiments were planted in 2023 on two different planting dates (June 28 and August 29). Seeds were planted on 1.02 m wide raised beds with two seed-lines and each bed was portioned into 6.1 m long plots with 0.61 m alleys. The experiment was laid out in a randomized complete block design (RCBD) with three replications. Seed planting was done using a tractor-pulled Precision Stanhay planter (Stanhay, UK), modified by Sutton AG Enterprises (Salinas, CA) to maneuver hand dropping of seeds for simultaneous planting into four seed-lines (two beds). After planting, plots were sprayed with an anti-crustant fertilizer to prevent crusting of the surface layer. Plots were irrigated by overhead sprinklers three times per week until thinning of seedlings (around 3rd- or 4th-week post-seeding) and then twice weekly for the duration of the experiment. Plots were thinned to 20–25 cm spacing between plants once plants were established (3–4 weeks after planting). Fertilizer was applied pre-planting (6–20-20 at 336.3 kg ha^−1^) and then at 4 and 6 weeks after planting (ammonium sulfate at 336.3 kg ha^−1^). Two herbicides, Kerb (3.5 L ha^−1^) and Prefar (9.4 L ha^−1^), were applied pre-emergence. During plant cultivation, mechanical weed control was performed with a tractor-mounted cultivator and by hand weeding. Fungicides were applied as needed later in the growing season to control downy mildew using Reason 500 (0.6 L ha^−1^), Aliete (2.2 kg ha^−1^), Revus (0.6 L ha^−1^), or Tanos DF (0.6 L ha^−1^). Insecticides were applied as needed for aphid control using Actigard (0.07–0.15 L ha^−1^) and Beleaf 50 (0.2 L ha^−1^). These insecticides are not labeled for thrips control.

#### Greenhouse experiments

Two greenhouse experiments were conducted in 2023, with one planted in April and another in September, to evaluate RILs for INSV resistance under controlled infection conditions. The 162 B×E RILs, two parents, and a susceptible check were sown in 192-well (8 × 24) plug trays filled with pasteurized (≈80 °C for 2.5 h) potting mix (premium potting mix, Sun Land Garden Product, Watsonville, CA) in each greenhouse experiment. After sowing, the plug trays were kept in the growth room at 20 °C with a 16-h photoperiod for four weeks until seedlings grew to ≈4 to 5-true leaf stage. A single seedling was allowed to grow per cell. While seedlings were growing in the growth room, they were supplemented twice (in week 3 and week 4, respectively) with nutrient solution (Peters Professional 20-20-20 general-purpose fertilizer; Everris NA Inc., Dublin, OH) prepared by dissolving 1 tsp per gallon of water. Four-week-old seedlings were then transplanted into pots (10.2 cm × 10.2 cm × 8.9 cm) filled with pasteurized potting mix and 1/4 tsp Osmocote® 14-14-14 (Everris NA Inc., Dublin, OH) per pot and placed inside a greenhouse containing spreader plants (INSV-susceptible plants sheltering a viruliferous thrips population; approximately 40 pots of spreader plants were placed per bench occupying 120 pots of experimental plants). In both greenhouse experiments, plant materials were divided into two treatment sets to induce INSV infection: 1) ‘Thrips only’ (T); a set of materials with INSV infection through thrips transmission, and 2) ‘Mechanical + Thrips’ (MT); a set of materials with infection by mechanical inoculation as described by Richardson et al. (2024) in combination with thrips transmission. In each greenhouse experiment, plant materials were arranged in a RCBD with three replicates. One plant of each RIL and three plants of each parent and a check were evaluated within each treatment set (T or MT) in each replicate. Climatic conditions of the greenhouse were set to 25 °C and a photoperiod of 16 h in both greenhouse experiments. Depending on cloudy or bright sunny days and supplemental LED lights, photosynthetically active radiation (PAR) fluctuated between ≈300 and 700 μmol m^−2^ s^−1^) in the greenhouse.

#### Disease assessment

In field experiments, 10 plants were marked within each plot in each replication and monitored for INSV infection. Disease assessment was conducted for each plant on a weekly basis beginning the 6th week (week 6) after planting to the 10th week (week 10). Disease severity (DS) assessment was performed by visually rating foliar symptoms of each plant using a 0–5 rating scale, with 0 indicating no INSV symptoms and 5 indicating complete plant death (Hasegawa and Del Pozo-Valdivia 2023). We also calculated disease incidence (DI) as the proportion of plants with a DS rating of 2 or more within a plot for each line. To ensure that visual symptoms were due to INSV and not caused by closely related tomato spotted wilt virus (TSWV), symptomatic plants were randomly sampled during middle phase of disease assessment (week 8) and tested for both INSV and TSWV using rapid lateral flow serological tests (ImmunoStrips; Agdia, Elkhart, IN). The presence of INSV was confirmed in the symptomatic plant samples, but no TSWV was detected in any sample in any experiment. In greenhouse experiments, disease assessments were conducted for each plant in the same manner as in the field except that the DI was not calculated due to the smaller number of plants. For field data, plot means of 10 plants were computed for each replication before statistical analysis. Area under the disease progress stairs (AUDPS) for both DS and DI data was calculated to combine the five weekly measurements (week 6–10) into a single value (Simko and Piepho 2012).

### Measurements of anthocyanin content and morphological traits

Anthocyanin content was measured using a portable Anthocyanin Content Meter (ACM-200plus; Opti-Sciences, Inc., Hudson, NH) that quantifies anthocyanins in intact plant leaves. The ACM-200plus measurements represent leaf anthocyanin content index (ACI) values, which are estimated based on optical absorbency at two wave bands, 530 nm and 931 nm (https://www.optisci.com/acm-200.html). ACI measurements were recorded on three random plants of each RIL and parents from the 2023 field experiment (August planting) by averaging three nondestructive readings taken from a young top leaf, a mid-aged leaf, and an older bottom leaf on each plant. ACI data were collected at three growth stages (the 5th, 7th, and 9th week after planting), with measurements taken on the same plants at each time point. Leaf color of the plants was also visually recorded using a 1–6 scale (1 = light green, 2 = green, 3 = light green with tinged red, 4 = green with tinged red, 5 = red with tinged green, and 6 = red). Furthermore, leaf glossiness (smoothness and reflective appearance) was assessed using a 1–3 scale (1 = relatively less glossy to 3 = glossier). ACI, leaf color, and leaf glossiness data were collected simultaneously from the same three plants, none of which exhibited visible INSV symptoms throughout the experiment. The rate of bolting was evaluated using a 1–6 scale (1 = rosette stage, 2 = expanded leaves, 3 = a bud beginning to emerge, 4 = a bud and internode emerged, 5 = multiple extended buds emerged, 6 = first flower emerged) as described in (Mamo et al. 2019).

### Statistical analysis

The descriptive statistics, correlation analysis, and analysis of variance (ANOVA) were performed using the R statistical packages (v. 4.4.2; R Core Team 2024). Initial ANOVA indicated that genotype, replication, and interaction effects were significant for the week-10 DS data. Therefore, for downstream analysis, fixed effects due to genotype (i.e., best linear unbiased estimators, or BLUEs) were extracted from a linear mixed model and used in place of the raw phenotypic values. A linear mixed-effects model was fitted with genotype as a fixed effect and replication as random effect to perform ANOVA and calculate BLUEs for each dataset separately, using the R package ‘lme4.’ Data across all experiments were analyzed with genotype treated as a fixed effect, while replication and experiment were treated as random effects, and ANOVA model was fitted with Kenward–Roger’s approximation. The 2023 field data from both June and August planting were not included in the combined or any other statistical analysis due to negligible disease pressure. The following models were utilized for calculating BLUEs:

For an individual experiment data: *Y* = *g*_*i*_ + *r*_*j*_ + *e*_*ij*_*.*

For combined experiment data: *Y* = *g*_*i*_ + *r*_*j*_ + *t*_*k*_ + *g*_*i*_: *t*_*k*_ + *e*_*ijk*_*.*

where *Y* represents the BLUEs of each genotype, *g*_*i*_ is the fixed effect of the *i*^th^ genotype, and *r*_j_ is the random effect of the *j*^th^ replication, *t*_*k*_*,* is the random effect of the *k*^th^ experiment, *g*_*i*_: *t*_*k*_ is the interaction between* i*^th^ genotype and *k*^th^ experiment, and *e*_*ij*_ and *e*_*ijk*_ represent the error terms.

Further, total variance was decomposed into genetic, experimental, their interaction, and error components considering all variable to be random. Heritability (*h*^*2*^) for each trait was calculated according to Hallauer et al. (1988):

For a single test (experiment): ${h}^{2}=\frac{{\sigma }_{g}^{2}}{{\sigma }_{g}^{2} + \frac{{\sigma }_{e}^{2}}{r}}$

For combined test (experiment): ${h}^{2}= \frac{{\sigma }_{g}^{2}}{{\sigma }_{g}^{2} + \frac{{\sigma }_{gt}^{2}}{n} + \frac{{\sigma }_{e}^{2}}{nr}}$

where ${\sigma }_{g}^{2}$ = genotypic variance, ${\sigma }_{gt}^{2}$ = genotype × test interaction variance, ${\sigma }_{e}^{2}$ = error variance, n = number of tests (experiments), and r = number of replications.

Statistical comparisons among parental lines, RILs, and the susceptible check were performed based on Dunn’s tests using the ‘FSA’ and ‘rcompanion’ packages following a significant Kruskal–Wallis test. Correlation analysis among morphological traits was performed using the Spearman method with ‘cor’ function and visualized using the ‘corrplot’ package.

### Stability test of RILs

Genotype main effect plus genotype-by-environment interaction (GGE) biplot analysis was performed to assess genetic stability of B×E RILs for INSV resistance. The analysis was performed using R package ‘metan’ to visualize the ‘DS Mean vs. Stability of INSV resistance’ plot as described in Richardson et al. (2024).

### Genotyping and linkage map construction

The 163 B×E RILs were genotyped with the lettuce 3 K DArTag panel (Lettuce_DArTag_Cornell_University 2.0), developed by Breeding Insight (RRID:SCR_026645) in collaboration with Diversity Arrays Technology (DArT) that employs a targeted genotyping approach viz. DArTag (Lin et al. unpublished). The lettuce 3 K DArTag genotyping platform generates 81 bp short sequences that were used for single nucleotide polymorphisms (SNPs) calling. Raw DNA sequence read alignment and variant calling were performed using a modified pipeline previously described (Pereira et al. 2021). Raw single-end FASTQ reads obtained from DArT were trimmed to remove adaptors and low quality reads using Trimmomatic (v0.39) with the parameters: SE -phred33 ILLUMINACLIP:TruSeq3-SE:2:30:10 LEADING:3 TRAILING:3 SLIDINGWINDOW:4:30 MINLEN:50 (Bolger et al. 2014). The trimmed reads were then aligned to the *L. sativa* V8 reference genome (Reyes-Chin-Wo et al. 2017; https://phytozome-next.jgi.doe.gov/info/Lsativa_V8) using the BWA-MEM algorithm (v0.7.17-r1188) (Li 2013). High-quality alignments with a mapping quality (MQ) score greater than 50 were retained for variant calling using samtools (v1.20) view -b -q 50. Variant calling was performed following the GATK (v4.3.0) best practices pipeline (Van Der Auwera et al. 2013). First, the GATK tool HaplotypeCaller was used (parameter: ERC GVCF) to produce gVCF files for each accession. These gVCF files were subsequently combined using CombineGVCFs, and genotypes were called using GenotypeGVCFs. SNPs were then extracted using the SelectVariants function. The resulting SNPs were then filtered using VariantFiltration with the following criteria: QD < 2.0, FS > 60.0, MQ < 40.0, MQRankSum < −12.5, or ReadPosRankSum < −8.0. An additional filter step was applied where only genotypes with a reference read depth of ≥ 1 or an alternative read depth of ≥ 2 were maintained for further analysis. A total of 1,866 polymorphic SNPs with a missing rate of ≤ 0.5 were retained for linkage map construction. Additionally, we included a set of 840 GBS (genotype-by-sequencing) markers from Mamo et al. (2019) for linkage map construction.

Overall, 2706 markers (1866 DArTag plus 840 GBS) were used to construct a combined linkage map with improved mapping resolution (Table S1). During the process of map construction, four RILs were flagged with elevated numbers of crossover/double crossover inconsistent with expectations (Fig. S2) and were thus removed from further linkage analysis. Moreover, nine additional RILs were excluded from the linkage analysis based on filtering threshold of > 0.1 missing data per individual. Subsequent filtering of markers with a missing rate of > 0.2, redundant genotypic information, or significant segregation distortion (Bonferroni-corrected p < 2.96 × 10^–5^) resulted in a final linkage map comprising 1598 high-quality SNPs across 150 B×E RILs. The final linkage map was constructed using OneMap v3.2.1 (Margarido et al. 2007; Taniguti et al. 2023). Pairwise recombination fractions were calculated between each pair of the 1,598 SNPs, which were used to cluster markers into nine linkage groups (LG) that correspond to the nine chromosomes in lettuce. For each linkage group, markers were ordered using the recombination counting and ordering (*RECORD*) approach. Genetic distances between the ordered SNPs were estimated using the hidden Markov model (HMM) multipoint approach, considering an observed genotype global error rate of 0.05 in the HMM emission function. The R package ‘LinkageMapView’ was used to visualize the genetic linkage map (Ouellette et al. 2018).

### QTL analysis

QTL analysis was performed using the R/qtl package (http://www.rqtl.org/; Broman et al. 2003). The recombination fraction was estimated using the ‘est.rf’ function. Genotype probabilities of the lines included in mapping were estimated using ‘calc.genoprob’ function (with an error probability of 0.001, a step-limit of 2 cM, and Kosambi map function) that utilizes HMM to estimate true underlying genotype between markers. For QTL analysis, BLUEs of the traits were used as phenotyping score for each RIL. Significant QTL was identified using ‘scanone’ function by implementing Haley–Knott regression (Haley and Knott 1992), for which the logarithm of odds (LOD) significance threshold was determined by a 1000 permutation test (*α* < 0.01). The percentage of the phenotypic variance explained (PVE) of the significant QTL was obtained by using ‘fitqtl’ function. The confidence intervals for each QTL were estimated using ‘lodint’ function that calculates the 1.5-LOD support intervals. Additive effect of the favorable allele at the significant QTL was calculated as (AA-BB)/2, representing half the difference in mean phenotypic values between genotypes homozygous for the favorable (AA) and unfavorable (BB) alleles at that locus.

### Candidate gene search

To identify candidate gene(s) associated with INSV resistance, all genes located within 1.5-LOD support intervals of the significant QTL were determined and annotated. Functional annotations of genes within this interval were obtained from *L. sativa* V8 genome assembly available on Phytozome (https://phytozome-next.jgi.doe.gov/info/Lsativa_V8). Candidate genes potentially involved in INSV resistance were then prioritized based on the frequency of gene functional annotations, with an emphasis on those potentially involved in plant defense responses.

### Enzyme-linked immunosorbent assay (ELISA) and total polyphenol assay

To detect INSV from plant samples, the double antibody sandwich (DAS)-ELISA was conducted according to manufacturer protocol (Agdia, Cat. SRA 20501). In the reaction plate, each sample was replicated twice, including the buffer control, healthy control, and INSV-positive control. ELISA reactions were measured using Bio-Tek Plate (Bio-Tek Instruments, Winooski, VT) at an absorbance wavelength of 405 nm. A sample was considered positive if the A_405nm_ value was 3 times greater than the means of healthy control samples. ELISA was performed on selected RILs and parental lines to assess INSV accumulation in leaf, crown, and root tissues, which were collected separately from the same plant for each line. Samples were collected from the field experiment planted in August 2023 (disease incidence was negligible that year) and the greenhouse experiment conducted in April 2023. As mentioned earlier, the field experiment was conducted to evaluate plants for INSV resistance under natural infection conditions, whereas the greenhouse experiment included two distinct inoculation treatments: T and MT.

Total polyphenol assay was conducted to assess variation in total polyphenol concentration in selected RILs and their parents. Leaf samples were collected from the field experiment planted in August 2023 at three different growth stages of plants, i.e., week 3, week 6, and week 10 after planting. Three plants of each line were randomly sampled, flash frozen in liquid nitrogen, and stored in −80 °C until further processing. The sampled plants were distinct from the ten plants marked for INSV symptom assessment in the field experiment and were sampled from the same plot where ELISA samples were collected. Total polyphenol extraction and quantification were conducted as described in Richardson et al. (2024).

## Results

### Phenotypic variation of INSV resistance in parental lines, RILs, and susceptible check

Weekly progression (week 6 to 10) of INSV symptoms in the parental lines, RILs from their cross, and the susceptible check ‘Defender’ are presented in three separate figures (Fig. S3, S4, and S5), each representing a different experiment. Across all experiments, ‘Defender’ exhibited high susceptibility with a rapid increase in INSV DS between week 6 and 10, with most plants nearly dead by the final week. ‘Reine des Glaces’ displayed moderate to high susceptibility, showing a steady rise in DS over the same period, whereas ‘Eruption’ consistently maintained low levels of DS throughout the duration of each experiment. The RIL population displayed a wide range of responses in each experiment, and significant ANOVA results confirmed that these differences were attributed to substantial genetic variation for INSV resistance (Table S2). Weekly disease progression data showed that the ‘week 10’ data are the most informative for statistical comparison and QTL mapping, as it captures the full development of symptoms across the plant’s maturity cycle and provide maximal phenotypic differentiation between resistant and susceptible lines.

**Fig. 1:**
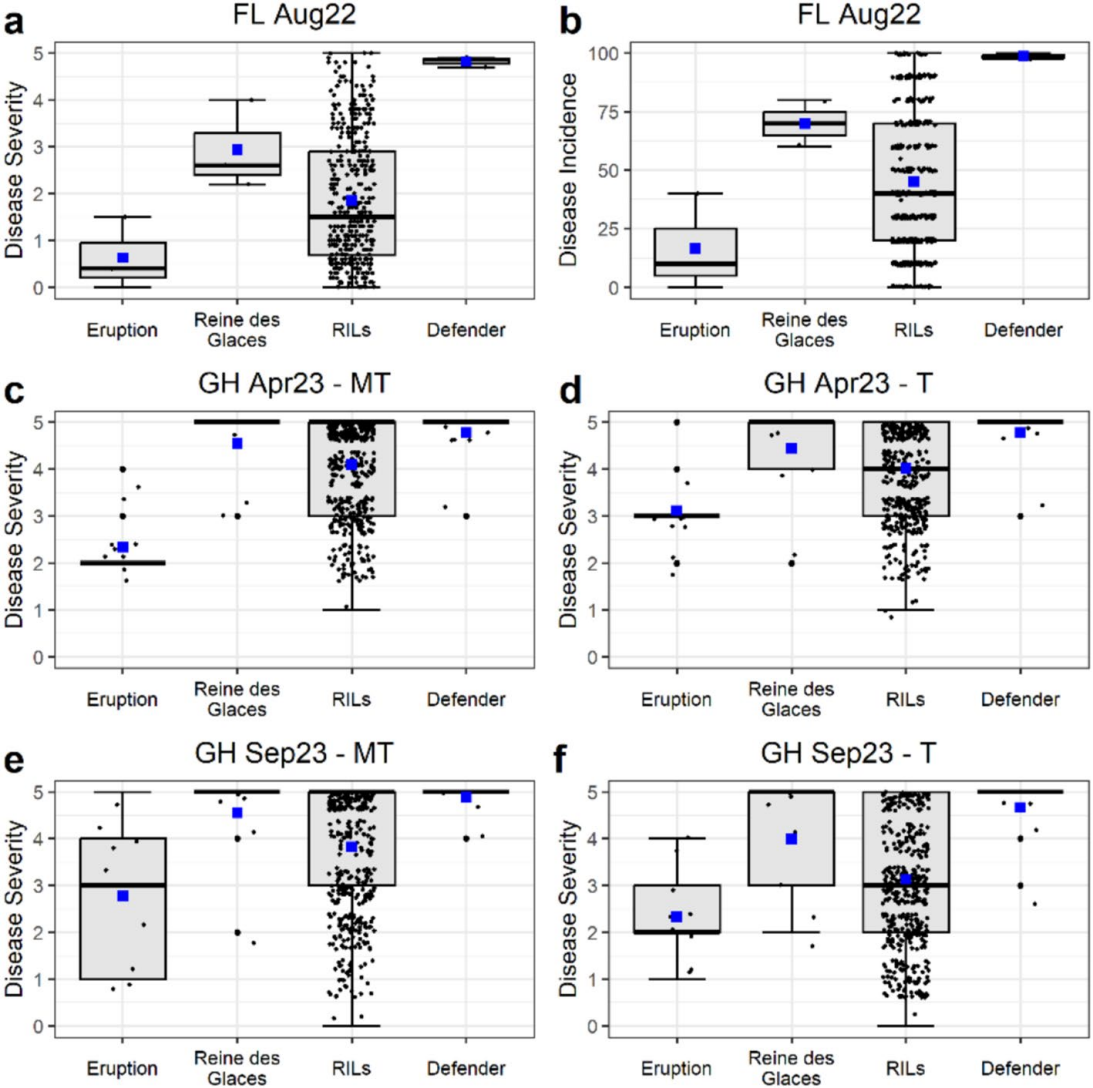
Variation in impatiens necrotic spot virus (INSV) resistance among lettuce genotypes across different field and greenhouse conditions. Boxplots show the disease severity (DS) and disease incidence (DI) at week 10 caused by INSV, in 162 recombinant inbred lines (RILs), their parents, and a susceptible check ‘Defender.’ The parents were ‘Eruption’ (resistant) and ‘Reine des Glaces’ (susceptible). DS was scored on a 0–5 scale and DI indicates proportion of INSV infected plants out of 10 plants evaluated per field plot. The small blue square within the box plot indicates mean value. **a** DS data—Field 2022; **b** DI data—Field 2022; **c** DS (Mechanical + Thrips) data—Greenhouse April 2023; **d** DS (Thrips only) data—Greenhouse April 2023; **e** DS (Mechanical + Thrips) data—Greenhouse September 2023; and **f** DS (Thrips only) data—Greenhouse September 2023

Analysis of week 10 data showed that the parental lines, ‘Reine des Glaces’ and ‘Eruption,’ were significantly different (*p* < 0.05) for INSV DS and DI in all experiments, including both greenhouse treatment sets (T and MT) used in INSV resistance evaluations (Fig. 1 and Table S3). ‘Eruption’ consistently showed a high level of partial resistance, maintaining a low levels of DS and DI, with a field mean DS of 0.63 and mean DI of 16.67%, and greenhouse mean DS values ranging between 2.33 and 3.11 across two different inoculation methods (T and MT). ‘Reine des Glaces’ showed moderate susceptibility in the field, with mean DS of 2.93 and DI of 70% and high susceptibility in the greenhouse, with a mean DS ranged from 4.0 (GH Sep23-T) to 4.56 (GH Apr23-MT and GH Sep23-MT). As expected, the susceptible check ‘Defender’ exhibited the most severe disease in all experiments, with a mean DS of 4.82 and DI of 98.7% in the field, and consistently high DS value ranging from 4.67 to 4.89 across all greenhouse experiments. The RIL population displayed substantial variation in disease responses (DS and DI) in both field and greenhouse experiments. The mean values of the RILs were between the parental lines (‘Reine des Glaces’ and ‘Eruption’) in each experiment.

Variance component analysis demonstrated that genetic factors account for substantial variation in RILs for INSV DS (Table S2). The results also showed that environmental factors can influence INSV DS; however, magnitude of genetic effects consistently remained strong. The heritability estimates for DS (using week 10 data) and AUDPS were moderate to high in individual experiments, ranging from 0.50 to 0.89 for DS, and 0.45 to 0.89 for AUDPS, with a heritability of 0.86 for combined data across all experiments for both DS and AUDPS.

### Correlation analysis between INSV DS, ACI, and other morphological traits

Correlation (Spearman’s rank) analysis was performed to assess relationships between INSV DS (week 10 DS of each experiment), ACI (at different growth stages), and other morphological traits (Fig. S6). DS ratings at week 10 showed a moderate positive correlation across experiments, with Spearman’s correlation coefficients (ρ) ranging from 0.51 to 0.66. Spearman’s ρ suggested that ACI had a negative, poor to no correlation with INSV DS (*ρ* = − 0.20 to −0.02). The rate of bolting, leaf color score, and leaf glossiness also showed similar relationships with INSV DS (inconsistent correlation, fluctuating between weak negative and weak positive; *ρ* = − 0.06 to 0.11 for the rate of bolting, *ρ* = − 0.15 to 0.07 for leaf color, and *ρ* = − 0.15 to − 0.03 for leaf glossiness).

### Genetic stability of the RILs for INSV resistance

**Fig. 2:**
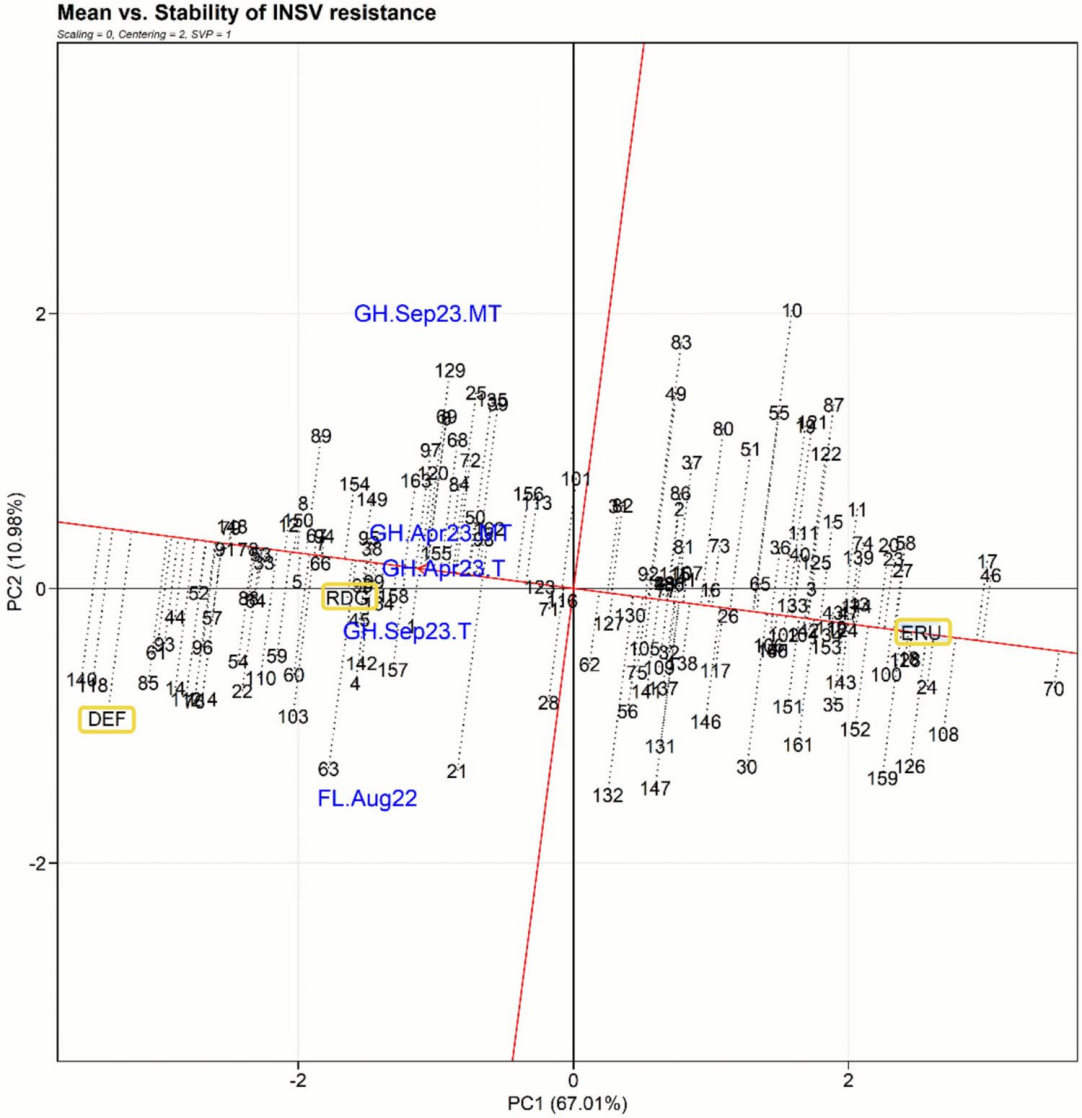
Genotype main effect plus genotype-by-environment interaction (GGE) biplot showing the mean disease severity (DS) and stability of impatiens necrotic spot virus (INSV) resistance for 162 recombinant inbred lines (RILs), the resistant parent ‘Eruption’ (ERU), the susceptible parent ‘Reine des Glaces’ (RDG), and a susceptible check ‘Defender’ (DEF). The biplot was constructed with the following parameters: scaling = 0 (data were not scaled), centering = 2 (data were centered by the means of each environment), and SVP (Singular Value Partitioning) = 1 (the singular value was partitioned into the genotype eigenvectors for visualizing the similarities among genotypes). Horizontal red line passing through biplot origin and marked with an arrow represents average environment axis (AEA) which points in the direction of increasing DS; genotypes located on the left side of AEA are more susceptible, while on the right side are more resistant. Stability of a genotype is determined by its distance from the AEA; those with a stable DS across experiments are located closest to the AEA line. The most desirable genotypes for a breeder have low DS and high stability, such as B×E16-070. Experiment names are shown in blue font and genotype names in black font. Abbreviations: FL.Aug22 = Field August 2022; GH.Apr23.MT = Greenhouse April 2023—Mechanical + Thrips; GH.Apr23.T = Greenhouse April 2023—Thrips only; GH.Sep23.MT = Greenhouse September 2023 – Mechanical + Thrips; GH.Sep23.T = Greenhouse September 2023—Thrips only. The numbers 1 to 163 represent B×E16-001 to B×E16-163

The GGE biplot (Fig. 2) illustrated a mean and stability analysis of the 162 B×E RILs along with parental lines and susceptible check for INSV DS (week 10) across five experimental conditions based on principal component analysis (PC1 and PC2). PC1 and PC2 are indicators of genotypic performance (mean DS) and stability of INSV resistance, each explaining 67.01% and 10.98% of the variation, respectively. Two RILs (e.g., B×E16-118 and B×E16-140) were positioned close to the susceptible check Defender (DEF) on the far-left side of the biplot, indicating higher susceptibility of these lines. In contrast, some other RILs (e.g., B×E16-017, B×E16-024, B×E16-046, and B×E16-070) were clustered near the resistant parent ‘Eruption’ (ERU) on the far-right side of the biplot, indicating they possess high levels of resistance with moderate to good stability. This GGE biplot effectively distinguished resistant and susceptible RILs and highlighted promising candidates, such as B×E16-070, for INSV-resistant germplasm release.

### Linkage map

**Fig. 3:**
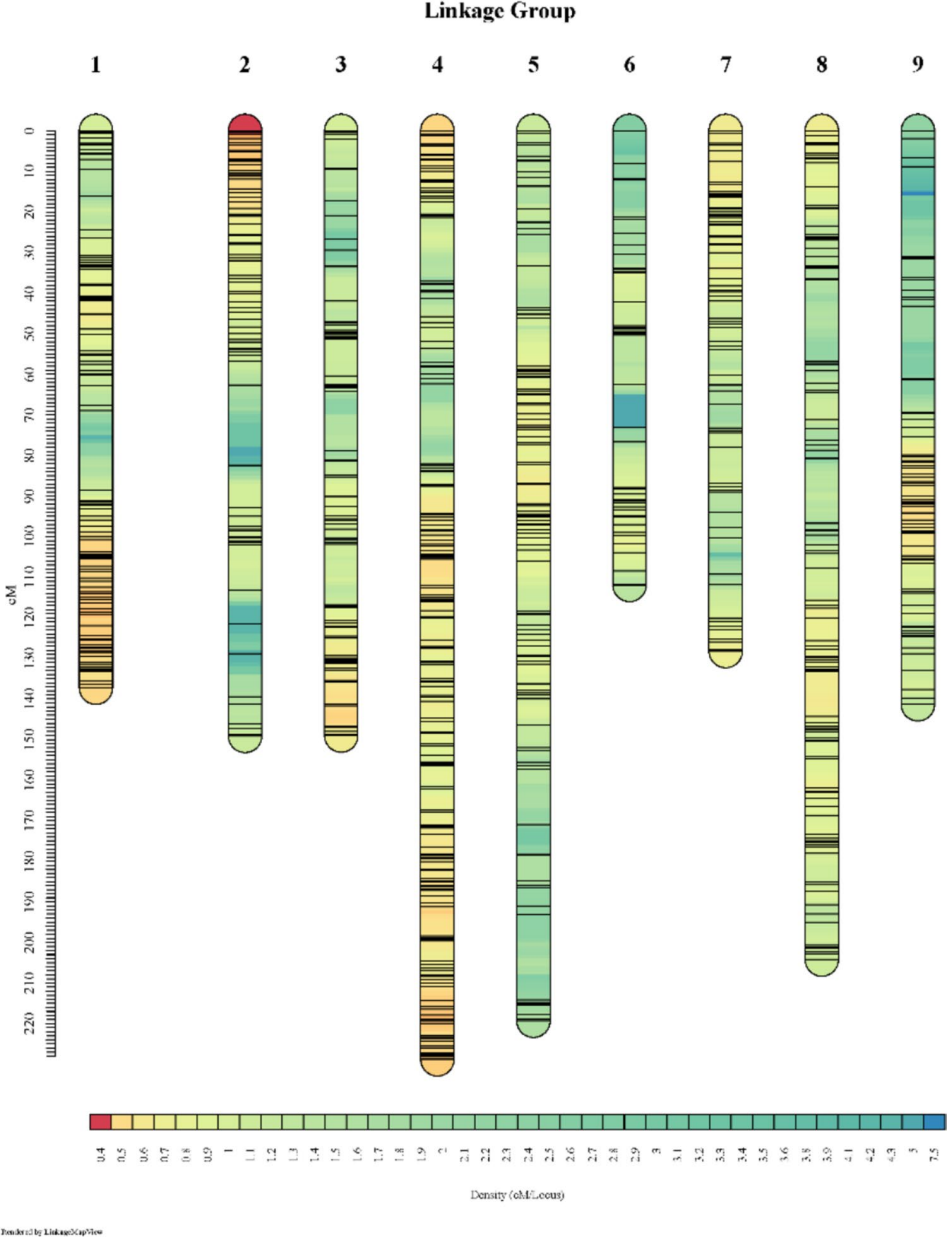
A mid-density genetic linkage map of 150 recombinant inbred line (RIL) population derived from ‘Reine des Glaces’ × ‘Eruption.’ The positions of the marker are indicated using a ruler shown on the left side

A total of 1598 SNPs (763 DArTag and 835 GBS) were mapped onto nine LGs corresponding to the nine chromosomes (Fig. 3 and Table 1). The linkage map spanned a total of 1469.09 cM genetic distance with an average marker density of 1.09 SNPs per cM. LG9 contained the widest gap in the map, spanning 22.28 cM (Table 1). Marker distribution on nine LGs varied between 82 on LG6 and 341 on LG4, with map length ranged from 111.94 cM (LG6) and 228.83 cM (LG4) (Table 1 and Table S4). All SNPs were assigned to LGs corresponding to their chromosomal positions on the *L. sativa* V8 reference genome, except for Chr9_6426948, for which the best match was found on LG3 (Lin et al. unpublished).

**Table 1:** Summary of the genetic linkage map constructed by using 1,598 single nucleotide polymorphism (SNP) markers generated by genotyping 150 recombinant inbred lines (Reine des Glaces × Eruption) using Diversity array technology targeted genotyping (DArTag) and genotyping-by-sequencing (GBS)

Linkage group (LG)	No. of markers			Map length (cM)	Maximum gap (cM)
	DArTag	GBS	Total		
1	81	107	188	137.17	19.69
2	70	87	157	149.11	19.68
3	72	80	152	148.97	14.6
4	165	176	341	228.83	19.83
5	101	100	201	219.34	21.1
6	46	36	82	111.94	12.18
7	71	68	139	128.22	9.04
8	102	109	211	204.23	20.22
9	55	72	127	141.28	22.28
Total	763	835	1,598	1,469.09	

### QTL associated with INSV resistance

**Fig. 4:**
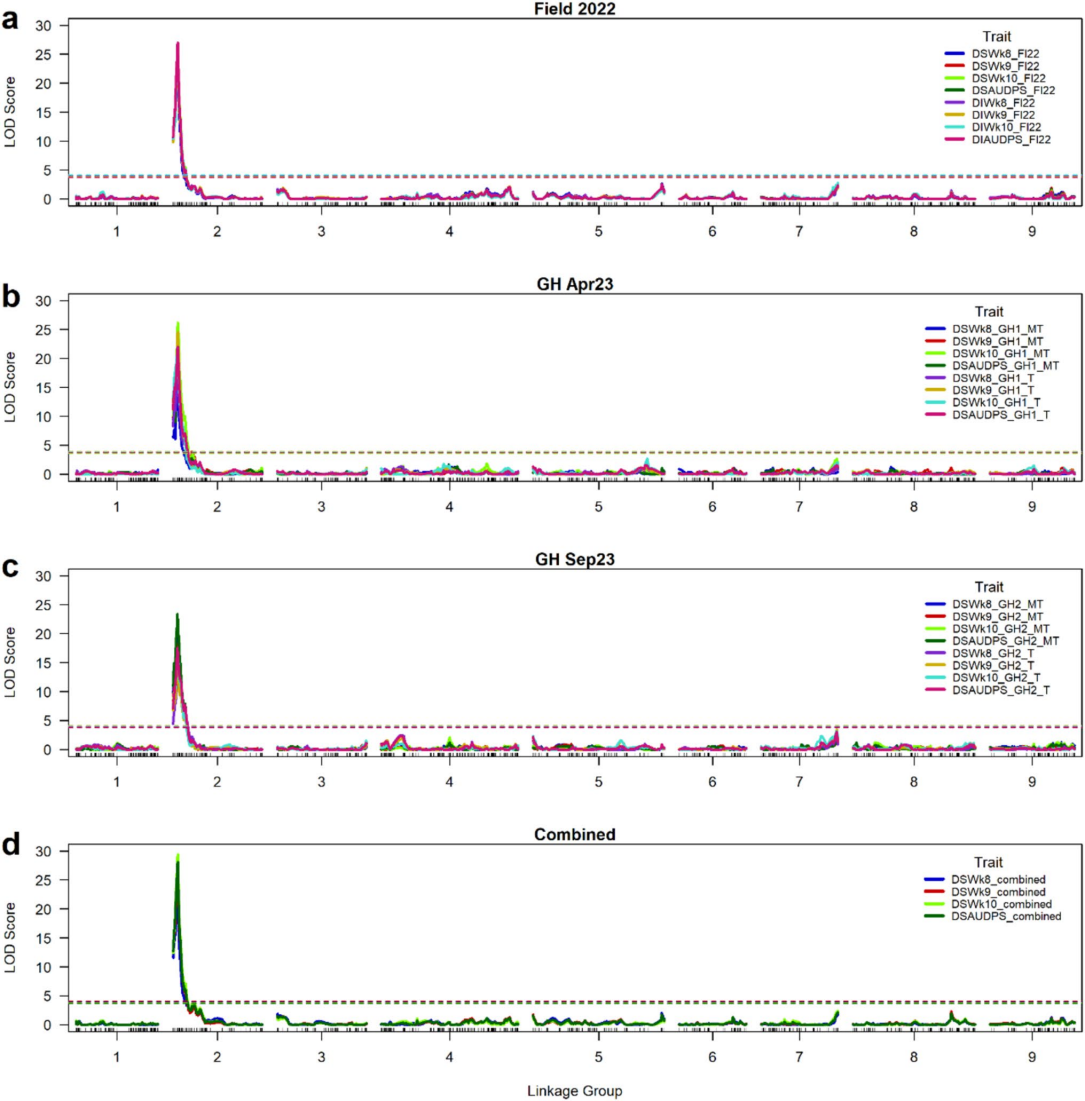
Quantitative trait loci (QTL) associated with impatiens necrotic spot virus (INSV) resistance identified in the ‘Reine des Glaces’ × ‘Eruption’ recombinant inbred lines (RILs) population. The mapping was performed using single nucleotide polymorphism (SNP) markers derived from Diversity array technology targeted genotyping (DArTag) and genotyping-by-sequencing (GBS) and best linear unbiased estimators (BLUEs) of phenotypic data. The y-axis shows the logarithms of odd (LOD) scores, and the nine lettuce linkage groups are shown along the x-axis. The horizontal dotted line indicates the LOD threshold for declaring a significant QTL. **a** ‘Field 2022’ represents data collected in Field August 2022 experiment; **b** ‘GH Apr23’ represents data collected in greenhouse April 2023 experiment; **c** ‘GH Sep23’ represents data collected in greenhouse September 2023 experiment; **d** ‘Combined’ represents combined data across all experiments

A single major QTL (*qINSV2.1*) associated with INSV resistance was repeatedly detected on LG2 (≈7–9 cM from the initial marker position) in all experiments, including the combined data across all experiments in the B×E RIL population (Table 2; Fig. 4). The LOD scores of the significant QTL ranged from 16.8 (GH Sep23-T dataset) to 26.7 (Field 2022 dataset) in individual experiments with LOD 29.4 for combined dataset of week 10 DS. Phenotypic variation explained (PVE) of this QTL was approximately 60% for the combined data. The LOD score for AUDPS was also high, ranging from 17.5 (GH Sep23-T dataset) to 25.2 (Field 2022 dataset), with 28.1 for the combined data. Favorable alleles, i.e., alleles that decreased INSV DS, contributed by ‘Eruption.’ This QTL was linked with the DArTag marker, Chr2_21135589, in most data sets. Two other DArTag markers (Chr2_19609747 and Chr2_16774385) and a GBS marker (Lsat_1_v5_g_2_8405) were also identified to be linked with this QTL in some datasets. All these markers are located within less than 1 cM (≈4.4 Mbp physical distance between Chr2_16774385 and Chr2_21135589).

**Table 2:** QTL *qINSV2.1* detected in the ‘Reine des Glaces’ and ‘Eruption’ RIL population across the field and greenhouse experiments that is associated with impatiens necrotic spot virus (INSV) resistance

Dataset	Trait	Peak Marker	Linkage Group	Map position (cM)	1.5-LOD support interval markers and their position		Threshold LOD (*P* < 0.01)	QTL LOD	PVE (%)	AE
					Upstream	Downstream				
Field 2022	DSWk8_Fl22	Chr2_21135589	2	8.29	Lsat_1_v5_g_2_1689 (7.03 cM)	Lsat_1_v5_g_2_89 (9.72 cM)	3.87	22.4	54.50	− 0.63
	DSWk9_Fl22	Chr2_21135589	2	8.29	Lsat_1_v5_g_2_1689 (7.03 cM)	Lsat_1_v5_g_2_89 (9.72 cM)	3.93	24.9	58.33	− 0.83
	DSWk10_Fl22	Chr2_21135589	2	8.29	Chr2_16774385 (7.29 cM)	Lsat_1_v5_g_2_89 (9.72 cM)	3.64	26.7	60.88	− 0.98
	DSAUDPS_Fl22	Chr2_21135589	2	8.29	Lsat_1_v5_g_2_1689 (7.03 cM)	Lsat_1_v5_g_2_89 (9.72 cM)	3.84	25.8	59.63	− 2.74
	DIWk8_Fl22	Chr2_21135589	2	8.29	Lsat_1_v5_g_2_1689 (7.03 cM)	Lsat_1_v5_g_2_89 (9.72 cM)	4.09	24.6	57.89	− 16.35
	DIWk9_Fl22	Chr2_21135589	2	8.29	Chr2_16774385 (7.29 cM)	Lsat_1_v5_g_2_89 (9.72 cM)	3.69	25.2	58.76	− 18.90
	DIWk10_Fl22	Chr2_21135589	2	8.29	Chr2_16774385 (7.29 cM)	Lsat_1_v5_g_2_89 (9.72 cM)	3.95	25.4	59.05	− 21.95
	DIAUDPS_Fl22	Chr2_21135589	2	8.29	Chr2_16774385 (7.29 cM)	Lsat_1_v5_g_2_89 (9.72 cM)	4.09	27.0	61.29	− 66.74
GH Apr23	DSWk8_GH1_MT	Chr2_21135589	2	8.29	Lsat_1_v5_g_2_1689 (7.03 cM)	Lsat_1_v5_g_2_89 (9.72 cM)	3.98	15.00	36.90	− 0.72
	DSWk9_GH1_MT	Chr2_21135589	2	8.29	Chr2_16774385 (7.29 cM)	Lsat_1_v5_g_2_89 (9.72 cM)	4.13	21.80	48.79	− 0.84
	DSWk10_GH1_MT	Chr2_21135589	2	8.29	Chr2_16774385 (7.29 cM)	Lsat_1_v5_g_2_89 (9.72 cM)	3.81	26.10	55.13	− 0.62
	DSAUDPS_GH1_MT	Chr2_21135589	2	8.29	Chr2_19609747 (7.64 cM)	Lsat_1_v5_g_2_89 (9.72 cM)	4.06	22.50	49.88	− 2.81
	DSWk8_GH1_T	Chr2_21135589	2	8.29	Lsat_1_v5_g_2_1689 (7.03 cM)	Lsat_1_v5_g_2_89 (9.72 cM)	3.77	17.30	41.21	− 0.72
	DSWk9_GH1_T	Chr2_21135589	2	8.29	Chr2_16774385 (7.29 cM)	Lsat_1_v5_g_2_89 (9.72 cM)	3.90	24.60	53.01	− 0.87
	DSWk10_GH1_T	Chr2_19609747	2	7.64	Lsat_1_v5_g_2_460 (5.17 cM)	Chr2_21135589 (8.29 cM)	4.00	21.90	48.95	− 0.59
	DSAUDPS_GH1_T	Chr2_21135589	2	8.29	Chr2_16774385 (7.29 cM)	Lsat_1_v5_g_2_89 (9.72 cM)	3.83	22.00	49.11	− 2.69
GH Sep23	DSWk8_GH2_MT	Chr2_19609747	2	7.64	Lsat_1_v5_g_2_460 (5.17 cM)	Chr2_21135589 (8.29 cM)	4.07	19.80	45.55	− 0.95
	DSWk9_GH2_MT	Chr2_19609747	2	7.64	Lsat_1_v5_g_2_1689 (7.03 cM)	Lsat_1_v5_g_2_89 (9.72 cM)	3.94	18.20	42.81	− 0.84
	DSWk10_GH2_MT	Lsat_1_v5_g_2_8405	2	7.64	Lsat_1_v5_g_2_1689 (7.03 cM)	Chr2_23498392 (10.42 cM)	4.13	17.40	41.39	− 0.63
	DSAUDPS_GH2_MT	Chr2_19609747	2	7.64	Lsat_1_v5_g_2_1689 (7.03 cM)	Chr2_21135589 (8.29 cM)	3.90	23.40	51.25	− 3.50
	DSWk8_GH2_T	Lsat_1_v5_g_2_8405	2	7.64	Lsat_1_v5_g_2_1689 (7.03 cM)	Chr2_21135589 (8.29 cM)	3.71	13.40	33.73	− 0.54
	DSWk9_GH2_T	Chr2_16774385	2	7.29	Lsat_1_v5_g_2_460 (5.17 cM)	Lsat_1_v5_g_2_89 (9.72 cM)	3.75	13.50	33.93	− 0.55
	DSWk10_GH2_T	Chr2_21135589	2	8.29	Lsat_1_v5_g_2_1689 (7.03 cM)	Lsat_1_v5_g_2_89 (9.72 cM)	4.02	16.8	40.30	− 0.64
	DSAUDPS_GH2_T	Lsat_1_v5_g_2_8405	2	7.64	Lsat_1_v5_g_2_1689 (7.03 cM)	Chr2_21135589 (8.29 cM)	4.00	17.5	41.57	− 2.10
Combined	DSWk8_combined	Chr2_21135589	2	8.29	Lsat_1_v5_g_2_1689 (7.03 cM)	Lsat_1_v5_g_2_89 (9.72 cM)	3.56	21.3	48.00	− 0.61
	DSWk9_combined	Chr2_21135589	2	8.29	Lsat_1_v5_g_2_1689 (7.03 cM)	Lsat_1_v5_g_2_89 (9.72 cM)	3.77	27.4	56.88	− 0.81
	DSWk10_combined	Chr2_21135589	2	8.29	Lsat_1_v5_g_2_1689 (7.03 cM)	Lsat_1_v5_g_2_89 (9.72 cM)	4.01	29.4	59.45	− 0.98
	DSAUDPS_combined	Chr2_21135589	2	8.29	Lsat_1_v5_g_2_1689 (7.03 cM)	Lsat_1_v5_g_2_89 (9.72 cM)	4.08	28.1	57.80	− 2.69

LOD, logarithm of odds; PVE, phenotypic variation explained by the QTL; AE, additive effect (negative sign indicates that allele that reduces INSV infection originates from ‘Eruption’)

**Fig. 5:**
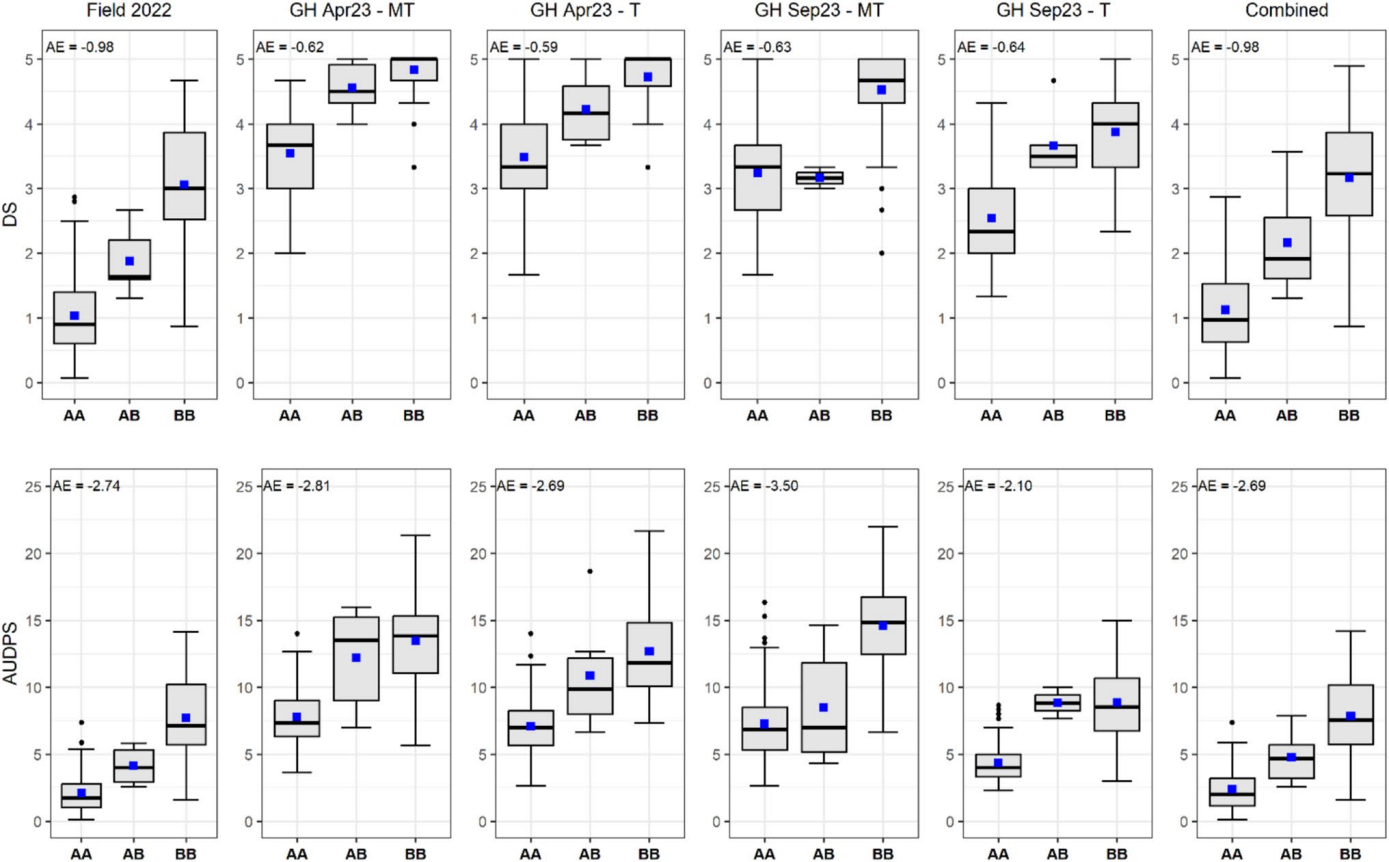
Allelic effects of *qINSV2.1* on impatiens necrotic spot virus (INSV) resistance across multiple experiments. Boxplots show the distribution of INSV disease severity (DS) on week 10 and area under disease progress stairs (AUDPS) among three genotypic classes (AA, AB, BB). Allele ‘A’ and ‘B’ are contributed by ‘Eruption’ and ‘Reine des Glaces,’ respectively. Each panel includes the additive effect (AE) of favorable allele (A) at the significant QTL, *qINSV2.1*. The small blue square within the box plot indicates mean value. Abbreviations of datasets: Field 2022 = Field 2022, GH Apr23-MT = Greenhouse April 2023—Mechanical + Thrips, GH Apr23-T = Greenhouse April 2023—Thrips only, GH Sep23-MT = Greenhouse September 2023—Mechanical + Thrips, GH Sep23-T = Greenhouse September 2023—Thrips only, Combined = Combined data across all experiments. Peak markers associated with *qINSV2.1* are Chr2_21135589 (DS: Field 2022, GH Apr23-MT, GH Sep23-T, and combined; AUDPS: Field 2022, GH Apr23-MT, GH Apr23-T, and combined), Chr2_19609747 (DS: GH Apr23-T; AUDPS: GH Sep23-MT), and Lsat_1_v5_g_2_8405 (DS: GH Sep23-MT; AUDPS: GH Sep23-T). Genotypic classes (AA/AB/BB) among recombinant lines are: 86/6/58 for markers Chr2_21135589, 84/6/60 for Chr2_19609747, and 74/2/62 (12 missing) for Lsat_1_v5_g_2_8405

We also assessed the genetic effect of *qINSV2.1* to determine its effect on INSV resistance using closely linked SNPs (Fig. 5). The B×E RILs were classified into three groups, with one group carrying the homozygous allele (AA) from ‘Eruption,’ another carrying the alternative homozygous allele (BB) from ‘Reine des Glaces,’ and a third group with the heterozygous allele (AB). Our results showed that RILs homozygous for the ‘Eruption’ allele (AA) had significantly decreased levels of INSV DS compared to those homozygous for the ‘Reine des Glaces’ allele (BB), with the heterozygous RILs exhibiting intermediate DS levels, indicating partial dominance of this QTL. The additive effect of the favorable allele at this QTL was ranged between −0.98 (Field 2022 and Combined data) and −0.59 (GH Apr23-T) for DS measured on the scale of 0–5. This indicates that genotypes carrying the favorable allele from ‘Eruption’ at *qINSV2.1* reduced INSV DS up to approximately one point on the rating scale relative to genotypes without the favorable allele.

### Candidate genes within the major QTL

**Fig. 6:**
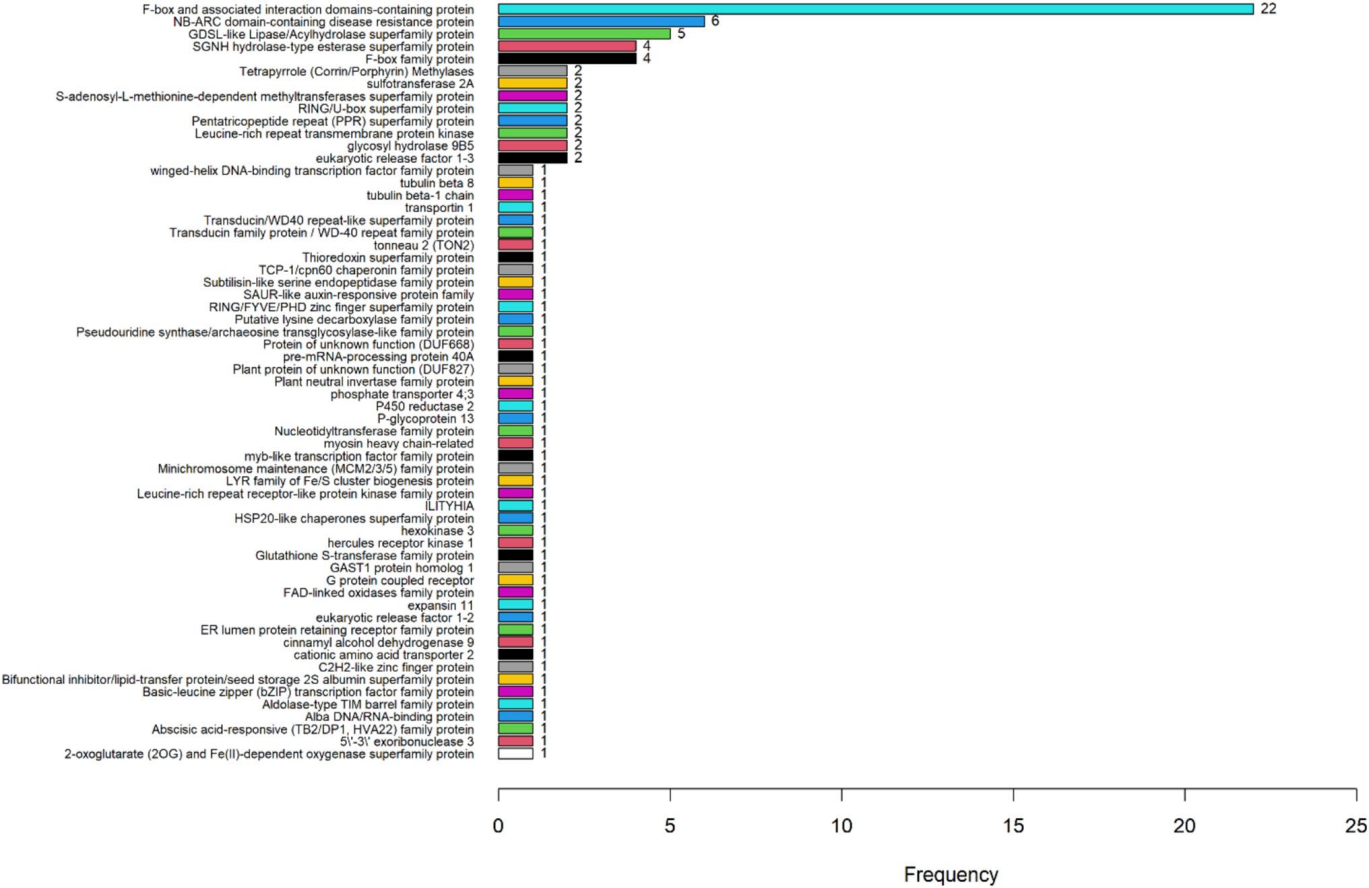
Frequency of annotated genes identified within the 1.5-LOD support interval of the major QTL (*qINSV2.1*) associated with impatiens necrotic spot virus (INSV) resistance in lettuce cultivar Eruption

The major QTL (*qINSV2.1*) was mapped within the 1.5-LOD support interval flanked by GBS markers Lsat_1_v5_g_2_1689 and Lsat_1_v5_g_2_89. To identify candidate gene(s) within this region, we expanded the search interval using neighboring DArTag markers (Chr2_14021920 and Chr2_23498392), which provide targeted sequence information encompassing the QTL region. Sequence alignment of these DArTag markers corresponded to an approximate 9.5 Mbp region on LG2, based on *L. sativa* V8 reference genome assembly. A total of 133 genes were annotated in this genomic interval (Table S5). Among these, genes encoding F-box and associated interaction domains-containing protein, NB-ARC domain-containing disease resistance protein, GDSL-like Lipase/Acylhydrolase superfamily protein, SGNH hydrolase-type esterase superfamily protein were the most frequent within the QTL region (Fig. 6 and Table S5).

### QTL for ACI

We detected two QTL for ACI on LG5 and LG9 (Fig. S7 and Table S6). Both ACI QTL were stable across different growth stages. The QTL on LG5 was consistently detected between marker interval Lsat_1_v5_g_5_3626 (43.5 cM) and Lsat_1_v5_g_5_1609 (52.0 cM) with LOD ranging from 5.9 to 8.1 and explained 16.6 to 21.9% variation in ACI at different growth stages. The QTL on LG9 was identified between marker interval Chr9_139766984 (108.0 cM) and Chr9_155478153 (113.9 cM) with LOD ranging from 11.6 to 12.3 and contributing 29.9–31.5% variation in ACI at different growth stages.

### Assessment of total polyphenol concentration (TPC)

**Fig. 7:**
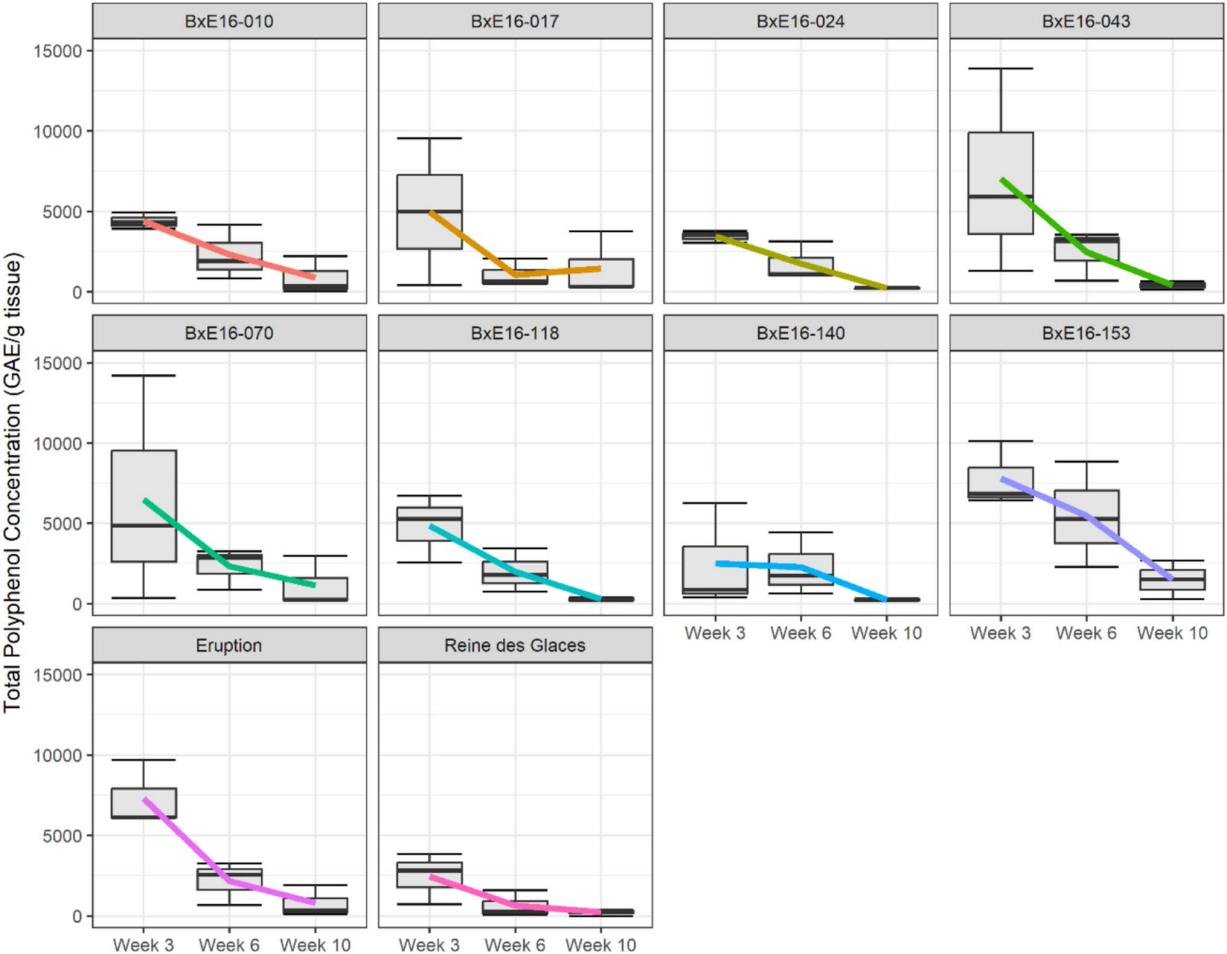
Pattern of total polyphenol accumulation in selected recombinant inbred lines (RILs) and their parents at different growth stages. As a brief recap, the overall mean disease severity (DS) scores across all experiments were 2.8 for B×E16-010, 2.0 for B×E16-017, 2.2 for B×E16-024, 2.6 for B×E16-043, 1.8 for B×E16-070, 4.9 for B×E16-118, 4.9 for B×E16-140, 2.8 for B×E16-153, 2.2 for ‘Eruption,’ and 4.1 for ‘Reine des Glaces’

TPC was assessed in parental lines (‘Reine des Glaces’ and ‘Eruption’) and eight selected RILs that exhibited either resistance or susceptible reactions to INSV (Fig. 7, Fig. S8, and Table S7). As a brief recap, the overall mean DS scores across all experiments were 2.2 for ‘Eruption,’ 4.1 for 'Reine des Glaces,’ 2.8 for B×E16-010, 2.0 for B×E16-017, 2.2 for B×E16-024, 2.6 for B×E16-043, 1.8 for B×E16-070, 4.9 for B×E16-118, 4.9 for B×E16-140, and 2.8 for B×E16-153 (Table S7). At early growth stages of plants (week 3), the variation in TPC among genotypes appears noticeable particularly with higher mean values in ‘Eruption,’ B×E16-153, B×E16-043, and B×E16-070 though these are not statistically significant that could be associate with type II error (Fig. S8). At later growth stages, i.e., by week 6 and especially week 10, TPC converged to lower levels with reduced variation (Fig. 7 and Fig. S8). Overall, TPC assays revealed that genotype-specific trajectories over different growth stages, with a few lines showing stronger early accumulation of TPC versus others such as ‘Reine des Glaces’ had low TPC throughout.

### Assessment of virus localization

ELISA-based detection of INSV from leaf, crown, and root tissues revealed a distinct tissue-specific virus accumulation pattern in the selected RILs and their parents (Table S8). ‘Reine des Glaces,’ the susceptible parent, showed high ELISA absorbance across all tissue types even when visual symptoms were not apparent in the field samples, with a high proportion of INSV-positive samples (8/8) for leaf, crown, and root tissue, confirming its systemic susceptibility to INSV. Susceptible RILs, such as B×E16-118 and B×E16-140, showed a similar pattern for the field samples. Greenhouse samples of these susceptible lines were not available for ELISA because all plants were dead by the time of sample collection. In contrast, the resistant parent ‘Eruption,’ showed low absorbance values with none to very few INSV-positive leaf samples, even though root and crown tissue were positive for INSV, indicating restricted virus accumulation and systemic movement within plant tissues. All the resistant RILs displayed a similar pattern across both field and greenhouse samples, apart from B×E16-043, where two out of three leaf samples collected from greenhouse experiment involving ‘Mechanical + Thrips’ inoculation tested positive for INSV.

## Discussion

Understanding the genetic basis of INSV resistance is important for breeding resilient lettuce cultivars, especially in regions like the Salinas Valley where the disease has intensified in recent years (Hasegawa and Del Pozo-Valdivia 2023; Simko et al. 2023a; Richardson et al. 2024). This study aims to advance the understanding of INSV resistance by identifying significant QTL, and by assessing mechanisms potentially contributing to resistance. For this purpose, we utilized the 162 B×E RIL mapping population to elucidate the genetic basis of INSV resistance. Substantial genetic variation for INSV resistance was evident among RILs, with the parental lines, ‘Eruption’ and ‘Reine des Glaces,’ displaying contrasting responses across all experiments. ‘Eruption’ consistently displayed low levels of INSV DS in all experiments, indicating a durable form of partial resistance consistent with earlier reports (Simko et al. 2018, 2023a; Richardson et al. 2024), whereas ‘Reine des Glaces’ exhibited moderate to high susceptibility. Significant genetic variation in RILs, and the clear phenotypic contrast between the parental lines for INSV resistance supports the suitability of this population for robust QTL identification, as recommended for effective trait dissection in biparental mapping populations (Collard et al. 2005).

Comprehensive phenotyping of mapping population under high disease pressure across field and controlled greenhouse experiments, combined with a mid-density linkage map, resulted in the identification of a major stable QTL (*qINSV2.1*) on LG2 associated with INSV resistance in lettuce cv. Eruption. Simko et al. (2023a) also reported two QTL on LG2, designated *qINSV2.1* and *qINSV2.2*, associated with INSV resistance in lettuce through GWAS, with at least 150 Mbp separation between the two. Among these, *qINSV2.1* was the most frequently detected across multiple environments, suggesting that this genomic region likely contains key genes contributing to INSV resistance. The major QTL identified in the present study is located within a similar genomic region as *qINSV2.1* reported by Simko et al. (2023a), suggesting that they may represent the same locus. Given this overlap, the major QTL identified in this study is referred to as *qINSV2.1* to maintain consistency with the previous nomenclature. No additional loci were detected in our study. Undetected minor loci, if present, may contribute to resistance, with their influence likely secondary to the major locus (*qINSV2.1*).

A recent patent (US9468186B2) describes a genetic determinant (referred as *insv1* by Simko et al. 2023a) which confers resistance to TSWV and/or INSV, also mapped on LG2. While both *qINSV2.1* (the QTL identified in this study) and the patented *insv1* are located on LG2, they appear to be distinct from each other. Physical map alignment indicated a separation of ≈20 Mbp between the two. Furthermore, *qINSV2.1* showed a pattern suggestive of partial dominance, as heterozygous individuals (AB genotype) exhibited intermediate resistance (Fig. 5), whereas *insv1* has been reported to be recessive in nature, conferring resistance only in homozygous state (Schut et al. 2016). These differences in genomic position and inheritance pattern strongly suggest that *qINSV2.1* and the patented *insv1* are separate loci. This distinction is important for breeders, as *qINSV2.1* is not subject to patent constraints and therefore represents a valuable and accessible genetic resource for breeders, which can be readily utilized in lettuce breeding programs to improve INSV resistance.

The B×E RIL mapping population, utilized in this study, was previously used to identify resistance QTL against lettuce drop caused by the soil-borne fungus *S. minor* (Mamo et al. 2019) and bacterial leaf spot caused by the bacterium *Xanthomonas hortorum* pathovar *vitians* (*Xhv*), formerly designated as *X. campestris* pv. *vitians* (*Xcv*) (Sandoya et al. 2019). While QTL for resistance to *S. minor* were not located on LG2, a major QTL for resistance to *Xhv* was mapped on LG2, in the same chromosomal region as *qINSV2.1*. This genomic region is known to harbor a large number of resistance genes against multiple pathogens (Simko et al. 2021). Nevertheless, the absence of *S. minor* resistance QTL on LG2 indicates their genetic independence of INSV resistance. Moreover, despite the physical proximity of QTL for *Xhv* and INSV resistance on LG2, it is unlikely that these two traits are controlled by the same locus, as resistance allele to INSV originates from ‘Eruption,’ whereas that to *Xhv* originates from ‘Reine des Glaces.’

Further, to gain insight into potentially relevant genes, we mined the 1.5-LOD support interval of *qINSV2.1*. This region contained 133 annotated genes, with those encoding F-box and associated interaction domains-containing protein, NB-ARC domain-containing disease resistance protein, GDSL-like Lipase/Acylhydrolase superfamily protein, and SGNH hydrolase-type esterase superfamily protein being most frequently represented. F-box proteins are integral components of the SCF (Suppressor of Kinetochore Protein 1-Cullin 1-F-Box) ubiquitin ligase complex and are involved in protein degradation pathways regulating diverse plant developmental processes, including hormonal signal transduction, secondary metabolism, and responses to both biotic and abiotic stresses (Lechner et al. 2006; Zhang et al. 2019). Notably, disruption of the interaction between F-box proteins with Suppressor of Kinetochore Protein 1 in *Nicotiana benthamiana* has been reported to enhance resistance to polerovirus (Lechner et al. 2006). Genes encoding NB-ARC domain-containing disease resistance protein were also among the most frequently represented within *qINSV2.1* region and are known to play important roles in regulating plant disease defense, including pathogen recognition and innate immunity (Van Ooijen et al. 2008; Wang et al. 2020). Similarly, genes encoding GDSL-like Lipase/Acylhydrolase superfamily proteins are also known to contribute to plant immunity. In *Arabidopsis*, GDSL lipase-like 1 (GLIP1) has been linked to both local and systemic immune responses against pathogens (Kwon et al. 2009). The SGNH hydrolase superfamily's GDSL-type esterase/lipase proteins (GELPs) were known to associate with plant growth and stress tolerance (Pahal et al. 2023). While these annotations provide preliminary leads into potential candidate genes, additional studies involving fine mapping and gene expression analysis may help elucidate their role in INSV resistance.

Breeders often investigate whether secondary traits can serve as indicators for primary traits, although such associations might also represent undesirable linkage or pleiotropic effects. In this study, we collected additional phenotypic data, including ACI, leaf color, bolting rate, and leaf glossiness to explore their potential association with INSV resistance. Our analysis revealed weak or no correlation (*ρ* = − 0.20 to − 0.02) between each of these traits and INSV DS. Previous studies reported that red leaf cultivars tend to exhibit less disease than green leaf cultivars (Richardson et al. 2024) and detected a weak negative correlation (*r* = − 0.28) between ACI and INSV incidence recorded over multiple years under natural infection (Simko et al. 2023a), suggesting a potential link between anthocyanins that produce red pigmentation in lettuce and INSV resistance. Current QTL analysis revealed two loci for ACI on LG5 (consistent with a previous study using the same mapping population by Mamo et al. 2019) and LG9 (a new QTL, likely detected in this study due to the higher resolution of the genetic linkage map). These two QTL colocalized with *Red Lettuce Leaves 2* (*RLL2*) and *anthocyanidin synthase* (*ANS*) genes, which are frequently identified in cultivated lettuce as key components of the anthocyanin biosynthesis pathway (Simko et al. 2023b). The lack of overlap between the ACI QTL and the major INSV QTL detected in this study suggests that INSV resistance in ‘Eruption’ and its derived lines are independent of anthocyanins accumulation. This is an encouraging sign for breeders, as the ‘Eruption’ source of resistance can be utilized for the development of INSV-resistant cultivars with a green leaf phenotype.

To gain further insights on physiological mechanisms contributing to INSV resistance, we assayed the TPC in parental lines (‘Reine des Glaces’ and ‘Eruption’) and in some selected RILs showing resistance or susceptible reaction to INSV. TPC assay results indicated some noticeable variation among genotypes at early growth stages (Fig. S8), with higher levels observed in ‘Eruption,’ B×E16-043 (green leaf), B×E16-070 (red leaf) and B×E16-153 (green leaf), while lower levels were noticed in ‘Reine des Glaces’ and B×E16-140 (green leaf). Although these differences were not statistically significant, they have revealed biologically meaningful trends. Genotypes with higher TPC were generally more resistant to INSV, including both red and green leaf lines, further supporting our conclusion that anthocyanins may not be the contributor to the resistance observed in ‘Eruption’ background. In contrast, the two lines with low TPC, both green leaf types, were among the most susceptible. These results suggest that, although anthocyanins are part of the phenolic compound family, other phenolics may be contributing to INSV resistance. Therefore, future studies focused on detailed profiling of phenolic compounds would be beneficial to identify those involved in conferring INSV resistance. Additionally, there was a clear trend of higher TPC during early plant development, which decreased significantly as plants approached maturity (Fig. 7). This temporal pattern suggests a polyphenol-mediated defense, and future studies may help determine whether this defense limits INSV infection by deterring the thrips vector or by directly restricting virus spread.

We also employed ELISA to assess tissue-specific INSV presence in both parents and the RILs that were used in the TPC assay to elucidate patterns of systemic virus movement within the plant. Leaf samples of the resistant parent ‘Eruption’ and resistant RILs were largely negative for INSV. However, crown and root tissues consistently tested positive even in resistant parent Eruption. In contrast, the susceptible parent ‘Reine des Glaces’ and the susceptible RILs tested positive for INSV across all three tissue types, leaf, crown, and root. This tissue-specific distribution of virus suggests that INSV resistance in lettuce may involve compartmentalization of viral accumulation, with roots and crowns acting as 'virus sinks' that restrict long-distance movement to shoots. A similar phenomenon has been reported in peanut infected with TSWV (Murakami et al. 2006). Our study provided some initial leads, yet future investigations should focus on targeted transcriptomic analysis across leaf, crown, and root tissues to fully understand how resistant lines restrict INSV movement in lettuce.

## Conclusions

We identified a major and environmentally stable QTL, *qINSV2.1*, conferring INSV resistance that was effective across different experimental conditions, including different inoculation methods. This QTL appears not to be linked with red pigmentation associated with anthocyanins, broadening its utility for developing green leaf cultivars with high levels of INSV resistance. Additionally, strong and stable resistance was observed in ‘Eruption’ and several RILs, e.g., B×E16-017 (red leaf), B×E16-024 (green leaf), B×E16-043 (green leaf), B×E16-046 (green leaf with tinged red), and B×E16-070 (red leaf). The identified QTL and these INSV-resistant lines offer valuable resources for breeding programs aiming to develop new INSV-resistant cultivars.

## Supplementary Information

Below is the link to the electronic supplementary material.

Captions and titles of the supplementary figures and tables (DOCX 35 kb)

Fig S1 (TIff 15520 kb)

Fig S2 (TIFF 313 kb)

Fig S3 (TIFF 18311 kb)

Fig S4 (TIFF 18311 kb)

Fig S5 (TIFF 18311 kb)

Fig S6 (TIFF 18311 kb)

Fig S7 (TIFF 29298 kb)

Fig S8 (TIFF 23438 kb)

Supplementary file1 (XLSX 1008 kb)

Table S1 (XLSX 21222 kb)

Table S2 (XLSX 13 kb)

Table S3 (XLSX 11 kb)

Table S4 (XLSX 847 kb)

Table S5 (XLSX 75 kb)

Table S6 (XLSX 79 kb)

Table S7 (XLSX 14 kb)

Table S8 (XLSX 14 kb)

## Data Availability

The data are contained within supplementary materials (Table S1 to S8). All relevant data, including raw and processed data for disease ratings, morphological traits, TPC, ELISA, and marker data are provided in the ‘Supplementary file1.’ Any additional query about data can be obtained from the corresponding authors upon reasonable request.
