# Supplementary material for: A major and stable QTL confers impatiens necrotic spot virus resistance in lettuce cv. Eruption
Source: Theor Appl Genet. 2025 Nov 28;138(12):312. doi: 10.1007/s00122-025-05058-9 (PMC12660355; doi:10.1007/s00122-025-05058-9)
Supplement: Supplementary file 1 — Captions and titles of the supplementary figures and tables (DOCX 35 kb) [file 122_2025_5058_MOESM1_ESM.docx]

**Fig. S1** Disease **s**ymptoms in lettuce plants infected with impatiens necrotic spot virus (INSV). **a-b.** Susceptible cv. Defender showing moderate to severe leaf necrosis in greenhouse conditions; **c.** Field-grown lettuce with dark brown necrotic lesions along the midrib; **d.** INSV-infected lettuce plants in a field plot; **e-f.** Greenhouse evaluation at week 10 showing cv. Reine des Glaces with variable symptom severity (e) and cv. Eruption exhibiting no visible symptoms (f).

**Fig. S2** Estimated number of crossovers and double crossovers for each of the 163 recombinant inbred lines (RILs) mapping population. RILs with an elevated number of crossovers and double crossovers are highlighted with ‘small circle’ in orange color.

**Fig. S3** Box plots showing weekly disease progression, disease severity (DS) and disease incidence (DI) incited by impatiens necrotic spot virus (INSV), in susceptible check ‘Defender’, two parents (‘Reine des Glaces’ and ‘Eruption’), and the 140 RILs in the field experiment conducted in 2022 under natural infections. The small blue square within the box plot indicates mean value.

**Fig. S4** Box plots showing weekly disease progression, disease severity (DS) incited by impatiens necrotic spot virus (INSV), in susceptible check ‘Defender’, two parents (‘Reine des Glaces’ and ‘Eruption’), and the 162 RILs in the greenhouse experiment conducted in April 2023 under ‘Mechanical + Thrips’ (MT) and ‘Thrips only’ (T) inoculation methods. The small blue square within the box plot indicates mean value.

**Fig. S5** Box plots showing weekly disease progression, disease severity (DS) incited by impatiens necrotic spot virus (INSV), in susceptible check ‘Defender’, two parents (‘Reine des Glaces’ and ‘Eruption’), and the 162 RILs in the greenhouse experiment conducted in September 2023 under ‘Mechanical + Thrips’ (MT) and ‘Thrips only’ (T) inoculation methods. The small blue square within the box plot indicates mean value.

**Fig. S6** Spearman’s rank correlation (ρ) between impatiens necrotic spot virus-disease severity (DS), anthocyanin content index (ACI), bolting rate, visual leaf color, and leaf glossiness (Smoothness and reflective appearance). Values in squares represent magnitude of correlation and negative signs indicate negative correlation. Abbreviations: ACI_23.Wk5 (ACI measurements on week 5); ACI_23.Wk7 (ACI measurements on week 7); ACI_23.Wk9 (ACI measurements on week 9); Bolt_mean (Bolting rate evaluated using a 1-6 scale; 1 = slow bolter to 6 = relatively quick bolter); Leaf_color_score (1 = light green, 2 = green, 3 = light green with tinged red, 4 = green with tinged red, 5 = red with tinged green, and 6 = red); Leaf_glossiness (1= relatively less glossy to 3 = glossier); DS.Wk10_Field22 (DS in 2022 field experiment conducted under natural infection); DS.Wk10_GHApr23.MT (DS in April 2023 greenhouse experiment conducted under ‘Mechanical + Thrips’ inoculation); DS.Wk10_GHApr23.T (DS in April 2023 greenhouse experiment conducted under ‘Thrips only’ inoculation); DS.Wk10_GHSep23.MT (DS in September 2023 greenhouse experiment conducted under ‘Mechanical + Thrips’ inoculation); DS.Wk10_GHSep23.T (DS in September 2023 greenhouse experiment conducted under ‘Thrips only’ inoculation)

**Fig. S7** Quantitative trait loci (QTL) associated with anthocyanin content index (ACI) in Reine des Glaces × Eruption recombinant inbred line (RIL) population at different growth stages. Abbreviations: ACI_Wk5 (ACI measurements on week 5); ACI_Wk7 (ACI measurements on week 7); ACI_Wk9 (ACI measurements on week 9).

**Fig. S8** Variation in total polyphenol concentration (GAE/g tissue) in selected recombinant inbred lines and their parents at different growth stages.

**Table S1** OneMap input file comprising marker data of the 163 lettuce B×E recombinant inbred lines (RILs) used for linkage map construction.

**Table S2** Variance components and heritability estimates obtained from the 162 F6:8 RILs (Reine des Glaces × Eruption) evaluated for INSV disease severity (DS) in individual and across all test conditions.

**Table S3** Comparison of disease severity (DS) and disease incidence (DI) means among parental lines, susceptible check, and recombinant inbred lines (RILs) in different experiments.

**Table S4** Marker data for the recombinant inbred line (RIL) population used in quantitative trait loci (QTL) analysis with genetic and physical map positions.

**Table S5** Genes identified in the 1.5-LOD support interval of the QTL peak and their functional annotations.

**Table S6** Overview of anthocyanins related quantitative trait loci (QTL) detected in the 'Reine des Glaces' and 'Eruption' recombinant inbred line (RIL) population.

**Table S7** Total polyphenol concentration (TPC; GAE/g tissue) at different growth stages and overall mean disease severity (DS) at week 10 of selected recombinant inbred lines (RILs) and their parents.

**Table S8** Summary of Enzyme-linked immunosorbent assay (ELISA) conducted on leaf, crown, and root tissues of selected recombinant inbred lines (RILs) and their parents.

**Supplementary File 1** Raw and processed datasets from this study, including disease ratings, morphological traits, total polyphenol concentration (TPC), Enzyme-linked immunosorbent assay (ELISA), and marker information.
