# Supplementary figures and images for: A major and stable QTL confers impatiens necrotic spot virus resistance in lettuce cv. Eruption
Source: Theor Appl Genet. 2025 Nov 28;138(12):312. doi: 10.1007/s00122-025-05058-9 (PMC12660355; doi:10.1007/s00122-025-05058-9)

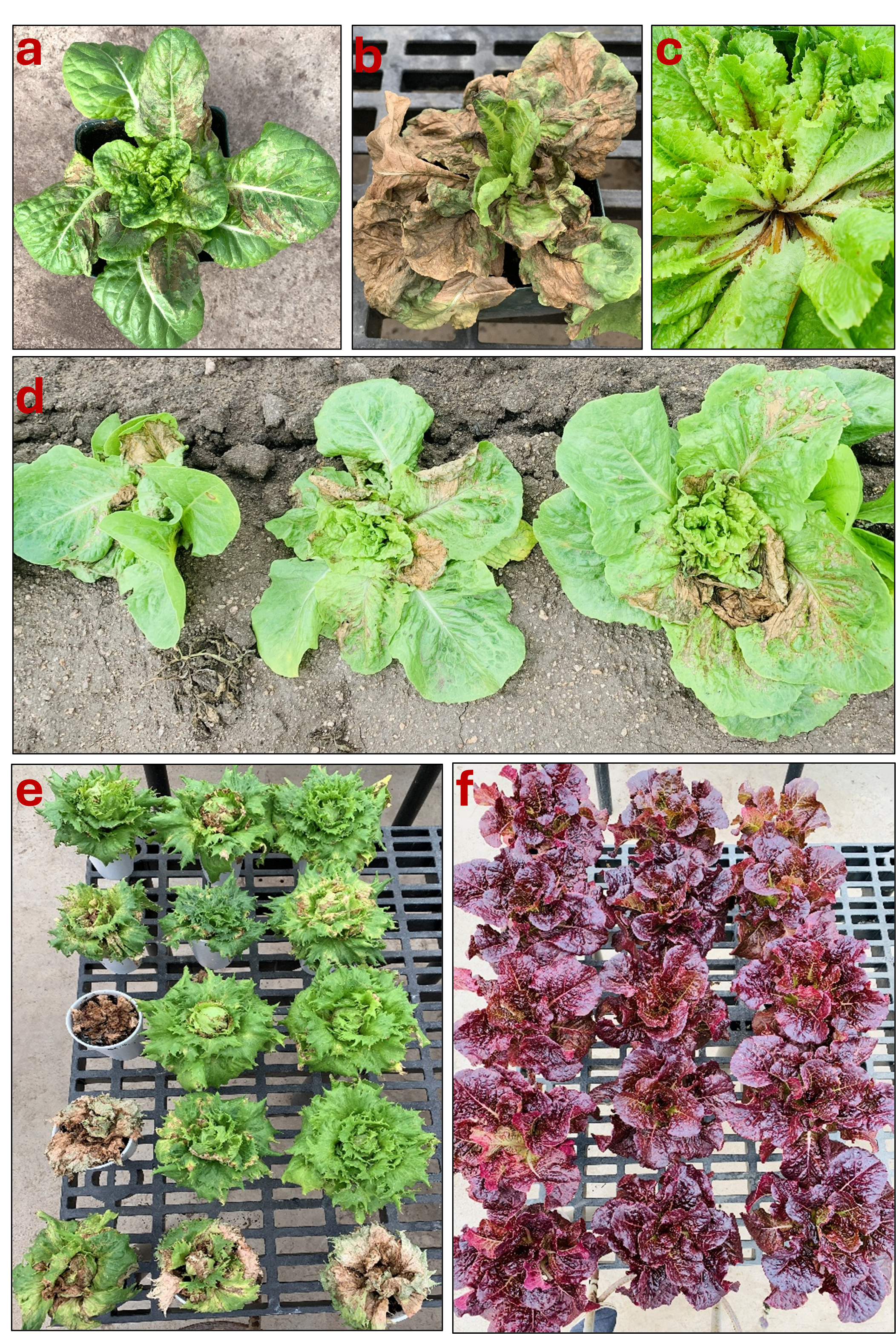

Supplement: Supplementary file 2 — Fig S1 (TIff 15520 kb) [file 122_2025_5058_MOESM2_ESM.tif]

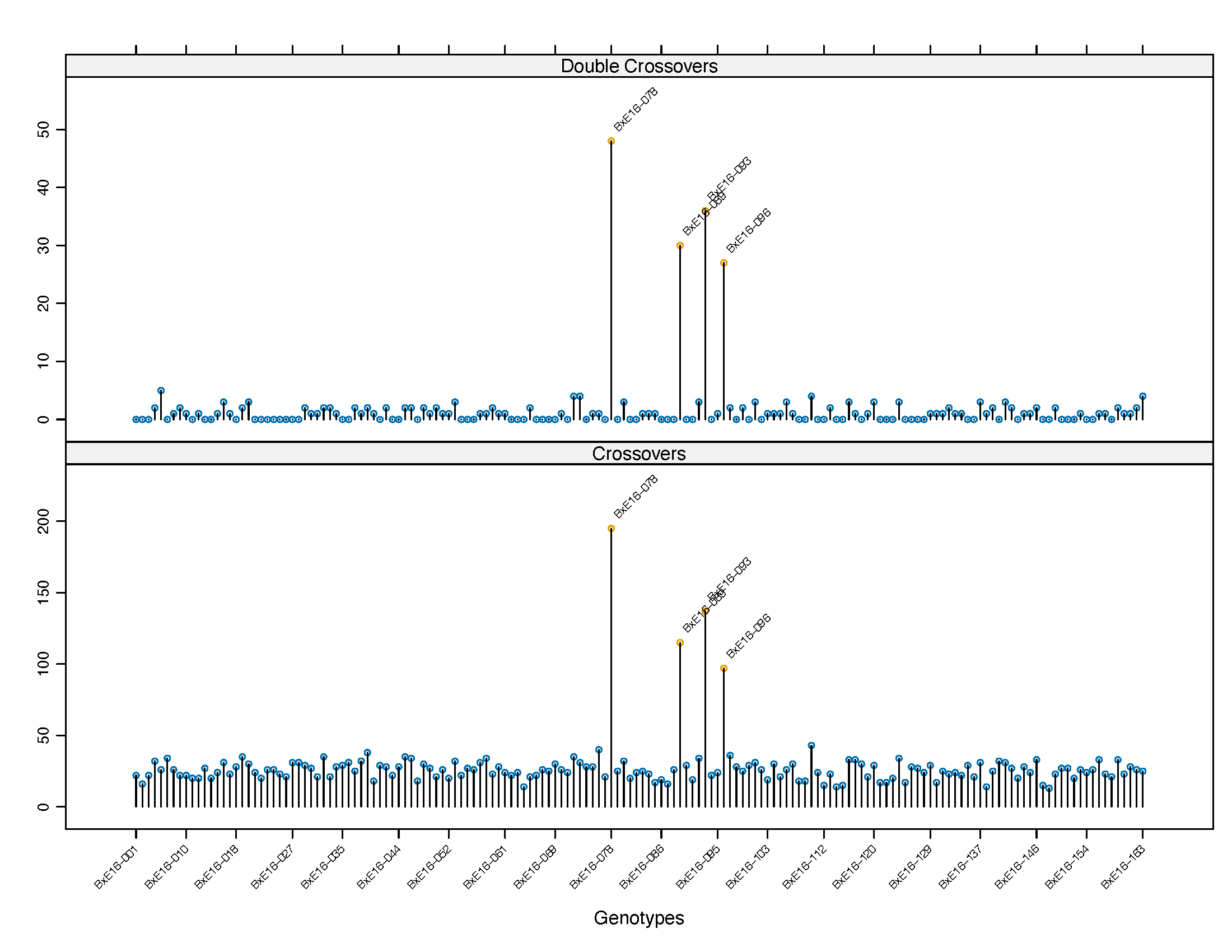

Supplement: Supplementary file 3 — Fig S2 (TIFF 313 kb) [file 122_2025_5058_MOESM3_ESM.tif]

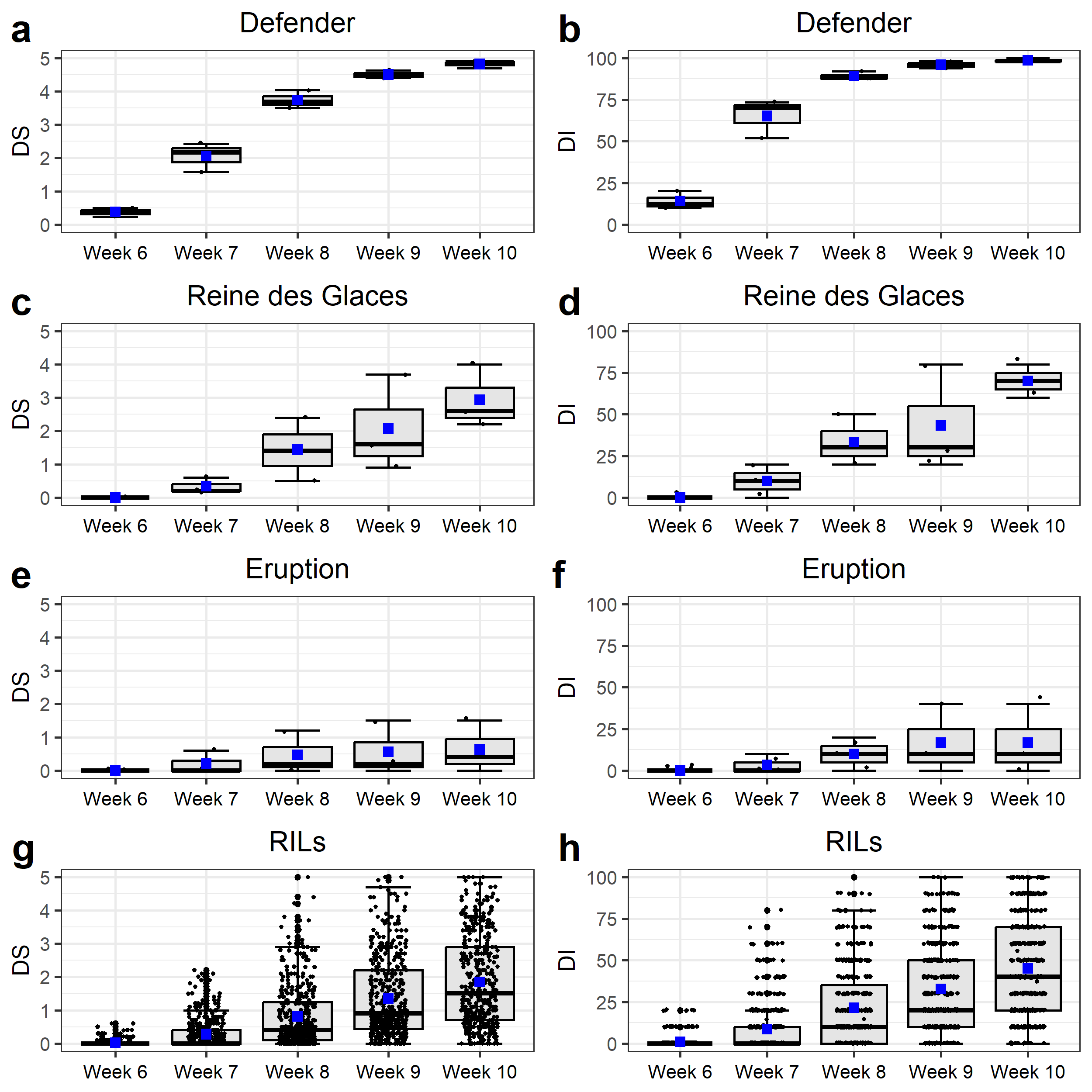

Supplement: Supplementary file 4 — Fig S3 (TIFF 18311 kb) [file 122_2025_5058_MOESM4_ESM.tif]

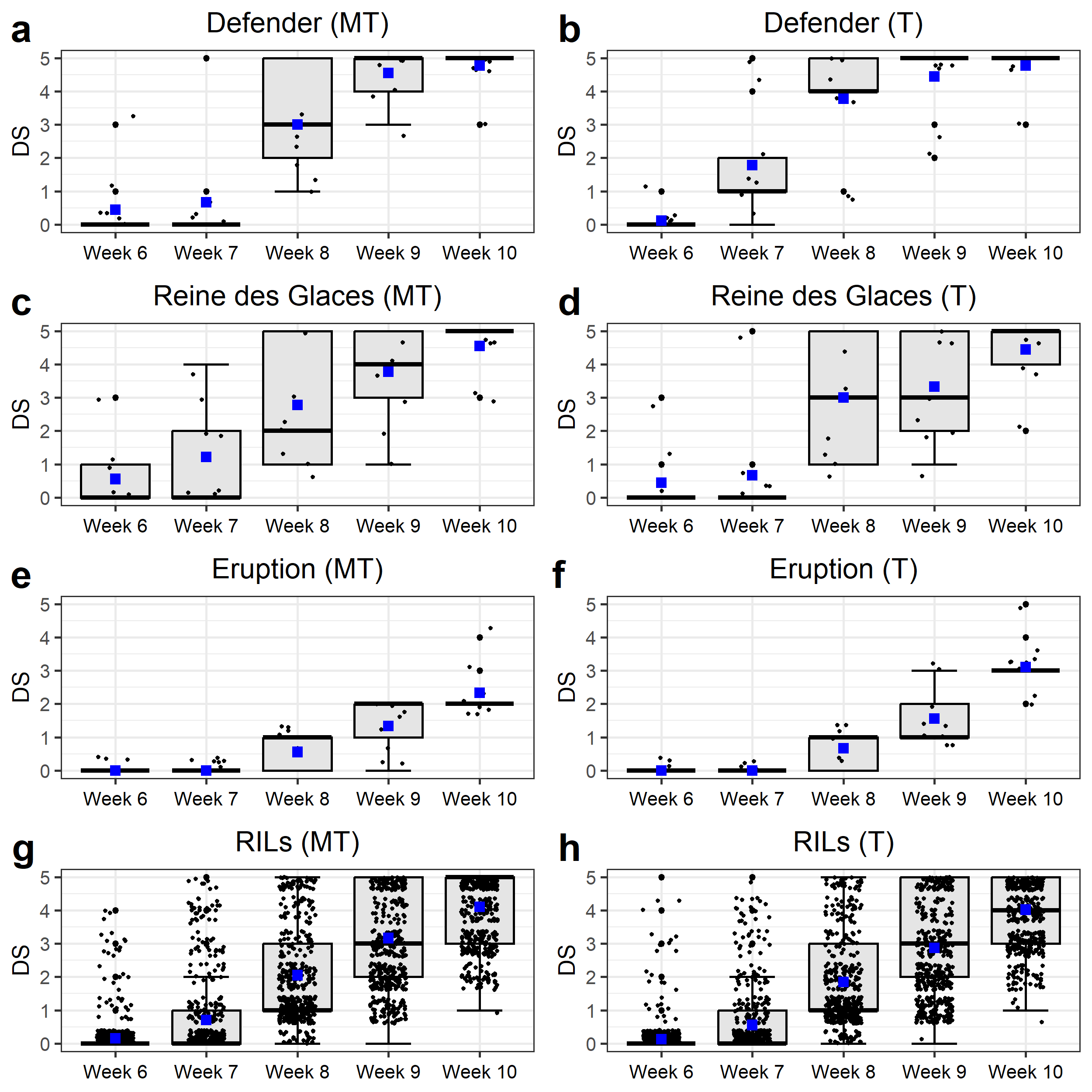

Supplement: Supplementary file 5 — Fig S4 (TIFF 18311 kb) [file 122_2025_5058_MOESM5_ESM.tif]

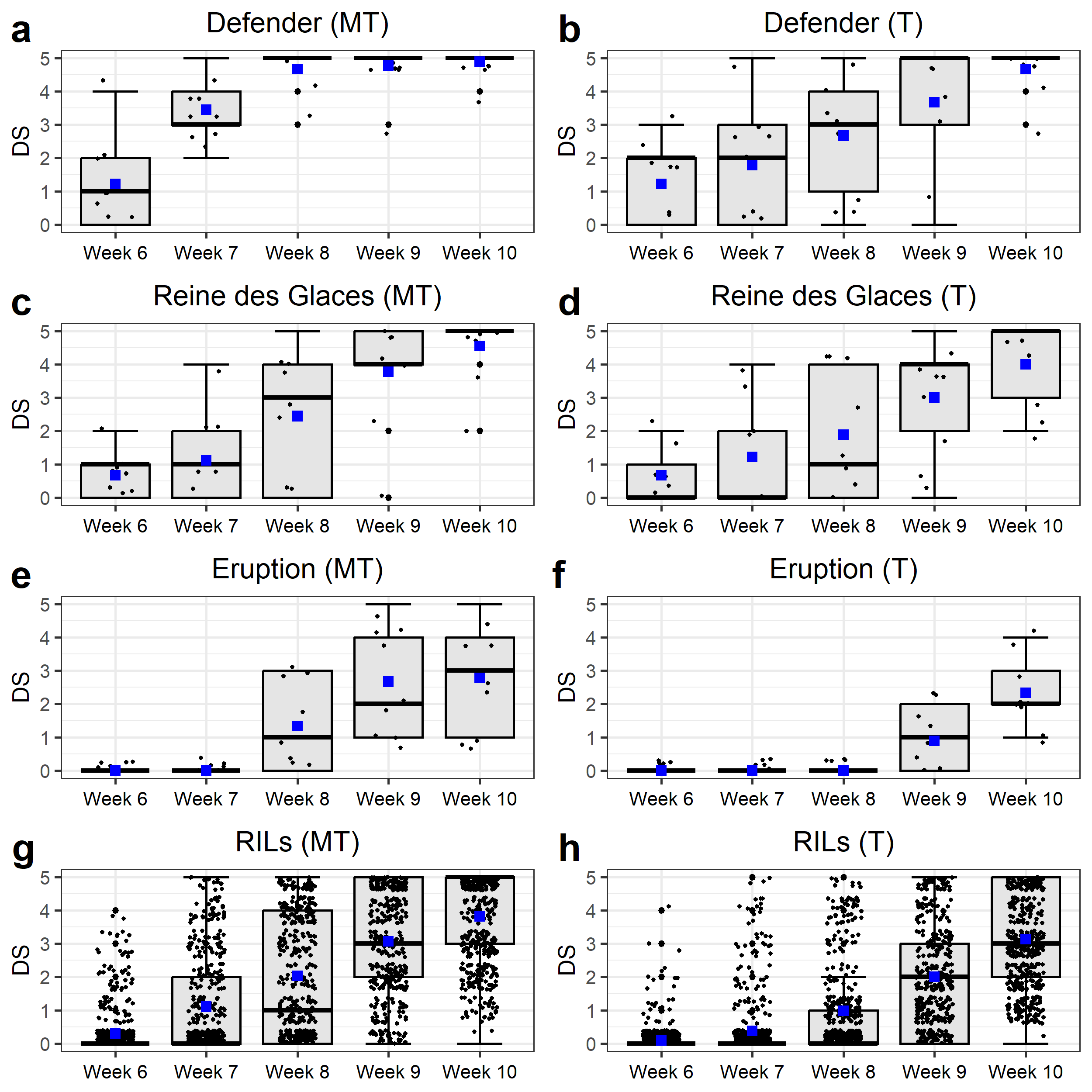

Supplement: Supplementary file 6 — Fig S5 (TIFF 18311 kb) [file 122_2025_5058_MOESM6_ESM.tif]

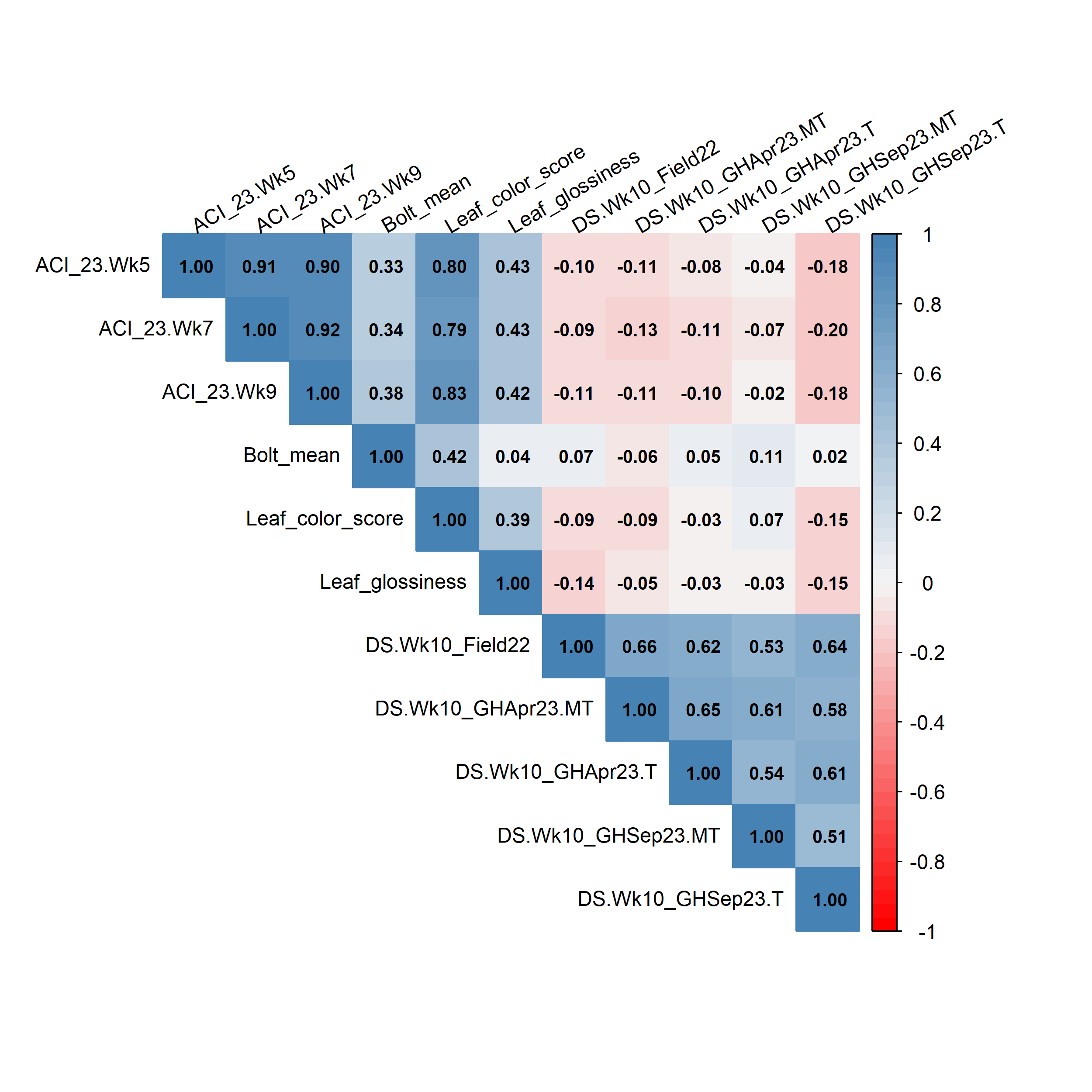

Supplement: Supplementary file 7 — Fig S6 (TIFF 18311 kb) [file 122_2025_5058_MOESM7_ESM.tif]

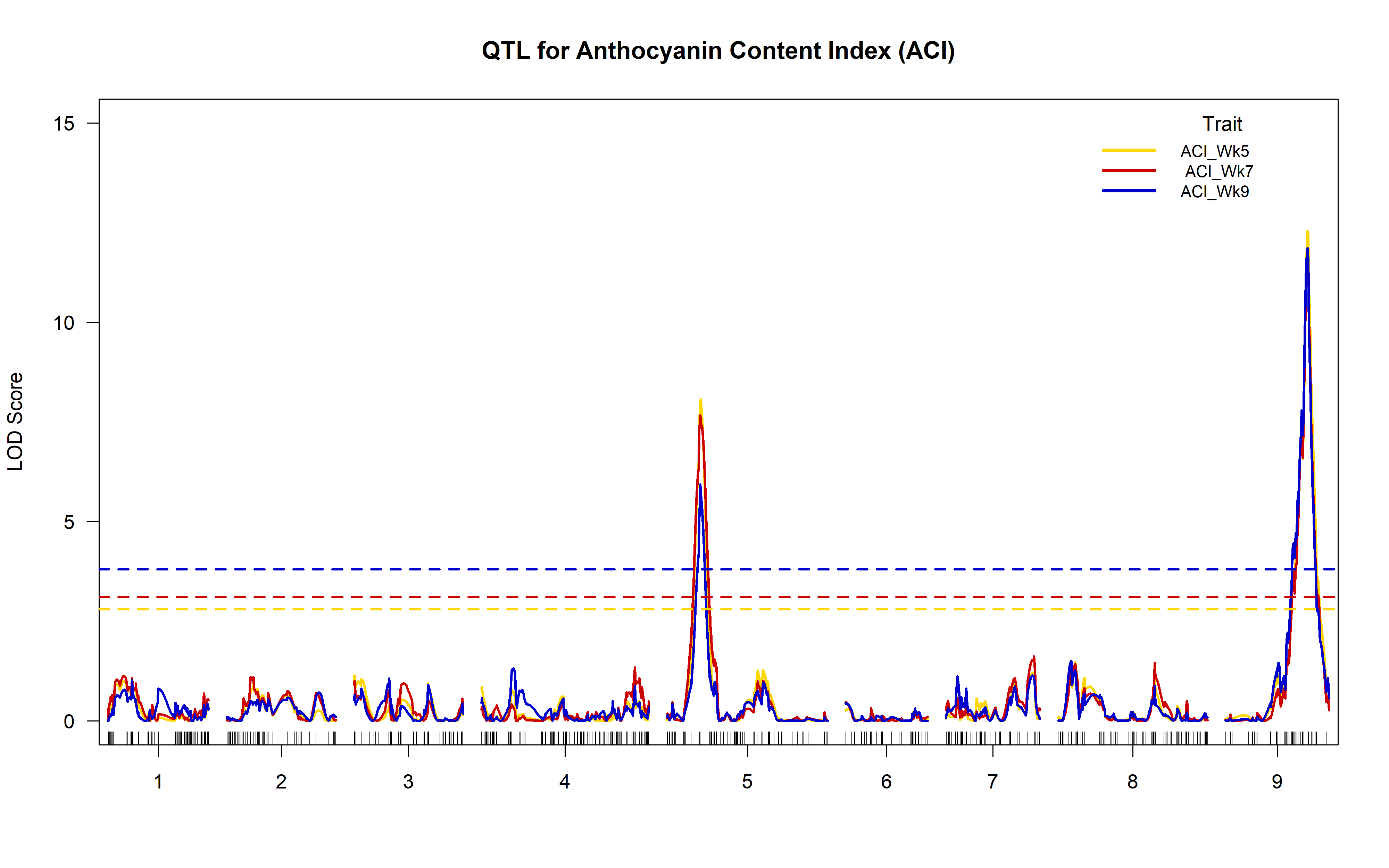

Supplement: Supplementary file 8 — Fig S7 (TIFF 29298 kb) [file 122_2025_5058_MOESM8_ESM.tif]

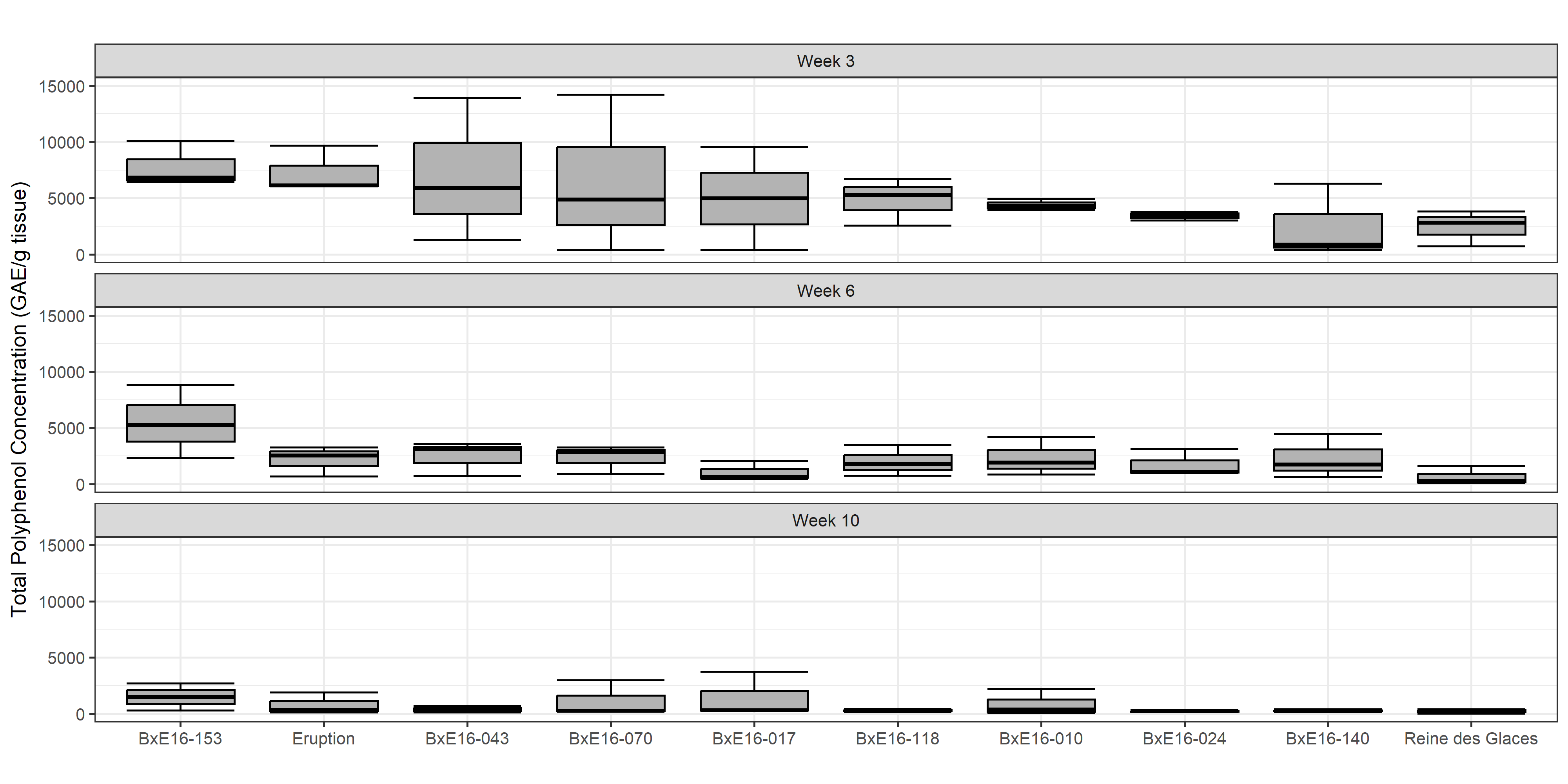

Supplement: Supplementary file 9 — Fig S8 (TIFF 23438 kb) [file 122_2025_5058_MOESM9_ESM.tif]
